# Model-based dietary optimization for late-stage, levodopa-treated, Parkinson’s disease patients

**DOI:** 10.1038/npjsba.2016.13

**Published:** 2016-06-16

**Authors:** Marouen Ben Guebila, Ines Thiele

**Affiliations:** 1 Molecular Systems Physiology, Luxembourg Centre for Systems Biomedicine, University of Luxembourg, Esch-sur-Alzette, Luxembourg

## Abstract

Levodopa has been the gold standard for Parkinson’s disease treatment for more than 40 years. Its bioavailability is hindered by dietary amino acids, leading to fluctuations in the motor response particularly in late-stage (stage 3 and 4 on Hoehn and Yahr scale) patients. The routine dietary intervention consists of low-protein (<0.8 g/kg) diets or the redistribution of daily protein allowance to the last meal. Computational modeling was used to examine the fluctuation of gastrointestinal levodopa absorption under consideration of the diet by (i) identifying the group of patients that could benefit from dietary interventions, (ii) comparing existing diet recommendations for their impact on levodopa bioavailability, and (iii) suggesting a mechanism-based dietary intervention. We developed a multiscale computational model consisting of an ordinary differential equations-based advanced compartmentalized absorption and transit (ACAT) gut model and metabolic genome-scale small intestine epithelial cell model. We used this model to investigate complex spatiotemporal relationship between dietary amino acids and levodopa absorption. Our model predicted an improvement in bioavailability, as reflected by blood concentrations of levodopa with protein redistribution diet by 34% compared with a low-protein diet and by 11% compared with the ante cibum (a.c.) administration. These results are consistent with the reported better outcome in late-stage patients. A systematic analysis of the effect of different amino acids in the diet suggested that a serine-rich diet could improve the bioavailability by 22% compared with the a.c. administration. In addition, the slower gastric emptying rate in PD patients exacerbates the loss of levodopa due to competition. Optimizing dietary recommendations in quantity, composition, and intake time holds the promise to improve levodopa efficiency and patient’s quality of life based on highly detailed, mechanistic models of gut physiology endowed with improved extrapolative properties, thus paving the way for precision medical treatment.

## Introduction

The gut wall is the first physiological barrier that encounters nutrients and xenobiotics absorbed orally,^1^ where food–drug interactions take place. These interactions are complex, as in the case of levodopa absorption and a protein-rich diet. Levodopa is the gold standard treatment in Parkinson’s disease (PD)^2^ and has a similar chemical structure as cyclic amino acids.^3^ Supplementing PD patients with levodopa restores the dopamine levels in the brain and prevents motor symptoms.^4^ It shares the same transporters with amino acids in the gut, brain, and kidneys, leading to competition that decreases its entry to target sites.^5^ Therefore, dietary recommendations are given to PD patients^6^ as levodopa bioavailability is influenced by diet.^7^ Low-protein diet (LPD) limits protein intake to a minimal amount for every meal (<0.8 g/kg of body weight). Another option is protein redistribution diet (PRD), where patients are recommended to take the daily protein allowance in the last meal. PRD has a better efficacy with lower off phenomena (e.g., akinesia) leading to improved life quality, especially at the latest stages of PD (3 and 4 on Hoehn and Yahr (HY) scale).^8^ The molecular mechanism of this improvement remains poorly understood. The recent identification of luminal and basolateral levodopa transporters in the human small intestine epithelial cell (sIEC) was a major step for a better evaluation of dietary recommendations. It has been shown that, in addition to luminal competition, the presence of amino acids in the basolateral side of enterocytes trans-stimulates the absorption of levodopa leading to a higher bioavailability of the drug.^9^

Computational modeling could be used to further elucidate the role of diet and levodopa absorption and to provide a basis for rational design of dietary recommendations. Such modeling should ideally (i) describe the levodopa kinetics in the gastrointestinal tract, as well as the other organs and (ii) consider sIEC-mediated uptake of levodopa and dietary amino acids.

For the first aspect, physiologically based pharmacokinetic (PBPK) modeling is an ideal computational approach because it describes with a set of ordinary differential equations cellular, tissue, and whole-body distribution of drugs. In particular, whole-body generic PBPK models have been described in the literature and used to investigate the effects of xenobiotics and mechanistically predict tissue concentrations of drugs.^10^ Moreover, organ and process-specific PBPK models have been developed, such as the advanced compartmental absorption and transit model (‘ACAT model’), for which *in vitro* parameters were used to estimate gastrointestinal absorption of drugs in human along seven small intestinal segments.^11^ An advantage of the PBPK modeling is that it captures the dynamics of the modeled system. However, it requires the availability or fitting of many parameters, which may not be always easy to be obtained and thereby limits the size and resolution of the described system. In contrast, the constraint-based reconstruction and analysis (COBRA) approach assumes the modeled biological system to be at a steady state, thereby allowing the description of large systems, such as cells and organs, at the molecular level, while not requiring complete knowledge about the model parameters.^12^

COBRA models are assembled based on genome annotations and biochemical data^14^ and subjected to physio-chemical, genetic, thermodynamic, and biological constraints.^15,16^ Recently, a genome-scale model of the enterocyte-specific metabolism (‘sIEC model’) has been published, describing known metabolic transformations and transport activities in sIECs.^16^ This model has been constructed on the basis of global human metabolic reconstruction^17^ which has been also recently extended for more refined metabolic content,^18,19^ as well as for a drug module.^20^

In this study, we (i) develop a combined multiscale PBPK–COBRA model and (ii) use it to investigate the complex spatiotemporal relationship between amino acids and levodopa kinetics and its impact on PD patients.

## Results

### Modeling intestinal absorption of levodopa using a combined PBPK–COBRA model

**Figure 1:**
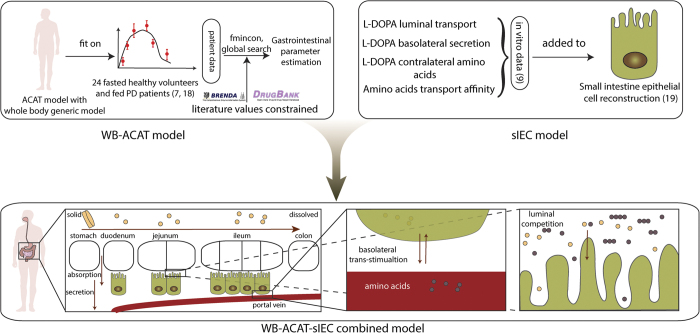
The multiscale PBPK–COBRA model used in this study. We combined a spatially and temporally refined PBPK model (deemed WB-ACAT) of the gastrointestinal tract with seven copies of a mechanistically accurate and detailed metabolic model of small intestinal epithelial cells (sIEC). COBRA, constraint-based reconstruction and analysis; PBPK, physiologically based pharmacokinetic; WB-ACAT, whole-body advanced compartmental absorption and transit.

**Figure 2:**
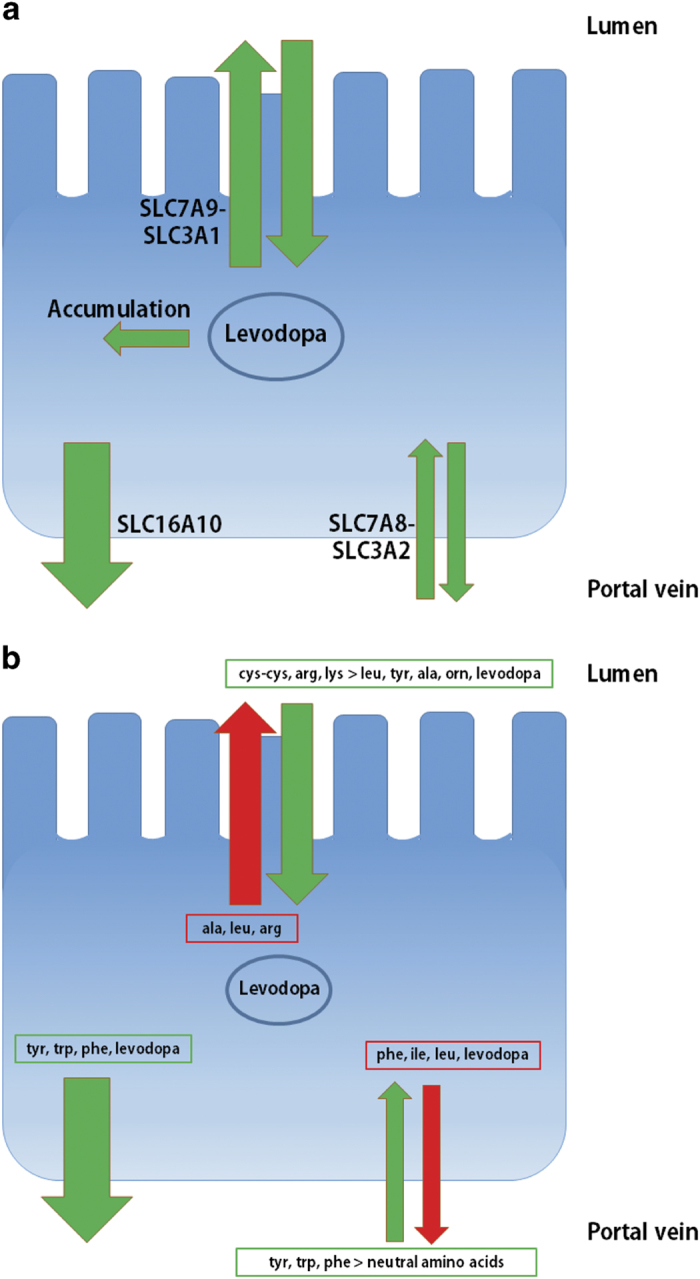
Levodopa and amino acids affinities for enterocyte transporters. (**a**) One transporter (antiporter) in the luminal side and two transporters (one antiporter and one uniporter) in the basolateral side are involved in the absorption and secretion of levodopa. (**b**) Amino acids are ordered by affinity for the transporter. Dibasic and neutral amino acids compete for the luminal uniporter. Aromatic amino acids uniporter is involved in the basolateral secretion of levodopa. The basolateral antiporter exchanges levodopa for amino acids. In both **a** and **b**, four fifths of levodopa is secreted through the basolateral uniporter and one fifth through the antiporter. Amino acids in the specific colored box go with the corresponding colored route.

To model the temporal, spatial, and metabolic effects of levodopa transport along the gastrointestinal tract, we developed a combined PBPK and COBRA model (Figure 1). This model combines the advantages of PBPK modeling through a comprehensive whole-body model with refined gastrointestinal absorption (WB-ACAT) model and of COBRA modeling through a mechanistically accurate and detailed small intestinal cell models (sIEC models), representing the metabolic functions of seven small intestinal segments (duodenum, two jejunum segments, and four ileum segments). The WB-ACAT model takes into account the dissolution and transit of a standard formulation of levodopa with respect to the pH and volume of each compartment, through the identified parameters of fasted and fed states. To ensure a biologically relevant set of parameters, the model was fit onto human data and constrained with values reported in the literature (Supplementary Figure S1B). In addition, the global search option with the constrained optimization algorithm (supplementary section parameter estimation) allowed obtaining a global minimum. The subsequent absorption, metabolism, and secretion of levodopa by enterocytes were captured by the sIEC models (Figure 2a) which were not added for the stomach and the colon as no absorption is assumed to take place in these two compartments. Both models were coupled using dynamic flux balance analysis (see Methods and Figure 1). The WB-ACAT-sIEC model was then used to identify patients in need of dietary recommendations and to provide mechanism-based optimized diet for PD patients. The internal metabolites of the sIEC were assumed to be in steady state, while levodopa had a concentration rate-of-change equal to the fluxes of the dynamical model through imbalanced exchange reactions.

### Dietary intervention is needed for HY 3 and HY 4 patients

**Figure 3:**
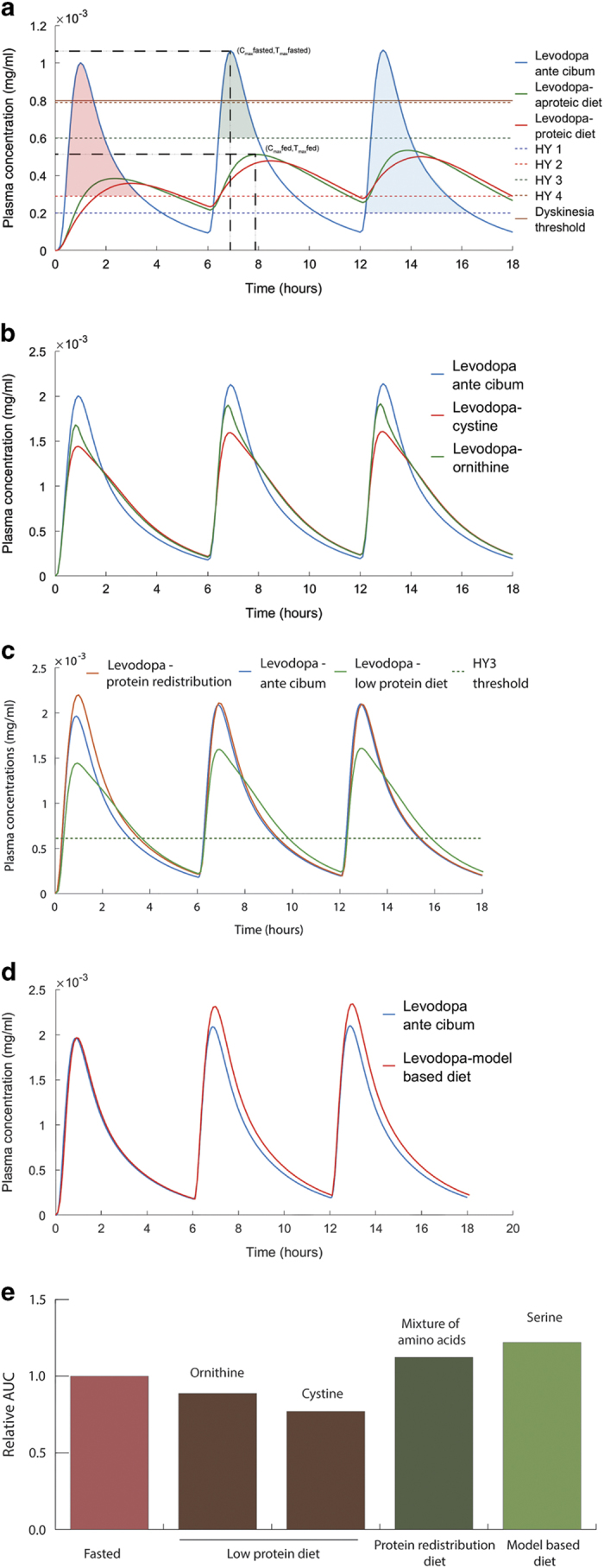
Predicted pharmacokinetics of levodopa under different dietary conditions. (**a**) a.c., proteic, and aproteic meals influence on the pharmacokinetic profile of levodopa. The efficacy threshold values for the different disease progression stages were plotted in dashed lines on Hoehn and Yahr scale (HY1 to HY 4). The more advanced the stage, the higher the levodopa concentration threshold. The AUC corresponding to HY1 in the fasted state is the blue area, HY 2 is the red area, and HY 3 is the green area (**b**) The pharmacokinetic profile of levodopa under LPD diet. (**c**) The pharmacokinetic profile of levodopa under PRD diet, LPD diet and a.c. administration. (**d**) The pharmacokinetic profile of levodopa with the model proposed serine-rich diet, which can increase the bioavailability of levodopa. (**e**) Relative variation of the AUC of levodopa under different dietary conditions in comparison to the fasted state. a.c., ante cibum; AUC, area under the curve; LPD, Low-protein diet; PRD, protein redistribution diet.

Using the multiscale PBPK–COBRA model, we addressed the question whether there is a particular subset of PD patients that would benefit the most by the recommended adjustment of dietary proteins.^7,8^ In particular, we investigated the pharmacokinetic profile of levodopa in a fasted state (Supplementary Figures S3 and S1C, supplementary section sequential fit on two occasions) and in a fed state with a concomitant administration of aproteic and proteic meal (Figure 3a). An ante cibum (a.c.) administration of 100 mg of levodopa every six hours was simulated (Figure 3a). Each disease state on HY scale has a therapeutic window, in which the efficiency of the treatment is optimal as reflected by the control of symptoms.^7^ A dose below the therapeutic window leads to persistence of the symptoms while a higher dose could trigger adverse effects, such as dyskinesia. Since the threshold concentrations of the therapeutic window have been determined with a 100 mg dose,^7^ the model was simulated at the specified doses, assuming that the parameters are dose independent. In fact, nonlinear behavior has been observed at very high, non-clinical doses.^21^ The resulting levodopa pharmacokinetic profile in the a.c. administration state showed a higher area under the curve (AUC) in the first HY stage (colored areas in Figure 3a). The decrease in AUC is inversely proportional to the disease stage. The simulation of the fed state with aproteic meal involved changing of physiological parameters (Table 1). The pharmacokinetic profile showed a decrease in the maximum concentration (*C*_max_) and a delay in the corresponding time (*T*_max_). With the concomitant administration of proteic and aproteic meals, the plasma concentration of levodopa stayed below the threshold of the therapeutic window of HY 3 and HY 4 patients and did not cover optimally the therapeutic window for HY 2. Based on this information, we concluded that dietary intervention would be most beneficial for PD patients in HY 3 and HY 4.

### PRD improves the bioavailability of levodopa over LPD

In later stages of PD, the reported superiority of PRD over LPD may be due to an increase in bioavailability of levodopa. To test this hypothesis, the pharmacokinetic profile of levodopa under both LPD and PRD was simulated. In the LPD setting, 0.8 g amino acids per kg body weight were administered *in silico* together with 200 mg of levodopa three times a day (t.i.d.) every 6 h. LPD assumes that levodopa dose is taken before the meal; hence, none of the physiological parameters (Table 1) were effective in the simulation. Since LPD and PRD are recommended in late-stage PD patients, with pronounced impairment of gastric emptying that can go up to 7 h,^22^ it is assumed that the last meal with <0.8 g/kg of body weight of proteins is still present in the small intestine. To account for the different affinities of amino acids for the luminal transporter and the subsequent competition with levodopa, cystine and ornithine were first simulated as these amino acids have the highest and lowest affinity (Figure 2b, Supplementary Table S3), respectively. The AUC above the efficacy threshold decreased by 11.24% for ornithine and by 22.91% for cysteine in comparison to the a.c. administration (Figure 3b).

**Figure 4:**
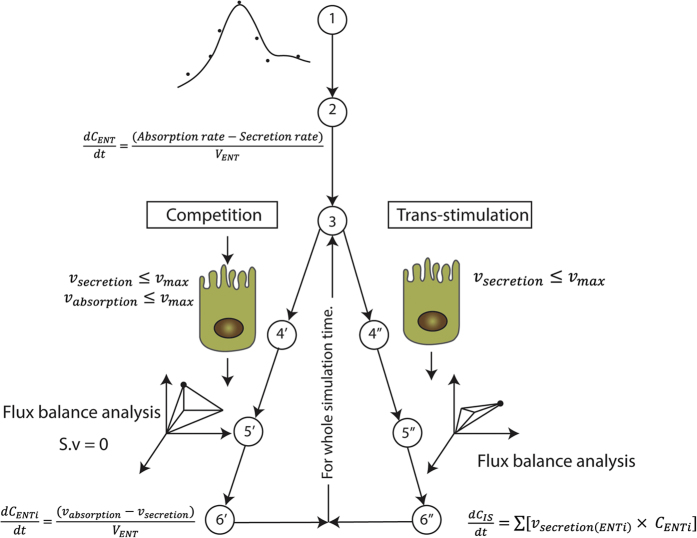
Multiscale modeling of the WB-ACAT-sIEC model for the absorption of levodopa by the small intestine. The key steps of the sequential coupling algorithm are illustrated. Step 1 (PBPK modeling): the physiological parameters of levodopa distribution were identified by fitting data from healthy individuals^45^ onto the WB-ACAT model. Step 2 (PBPK modeling): the WB-ACAT model, together with the obtained parameters, was simulated for one-time step. Steps 3 (PBPK modeling): the flux values were retrieved, depending on the competition or trans-stimulation mode, for absorption and secretion reactions/metabolites that are in common between the WB-ACAT and the sIEC models. Step 4 (COBRA modeling): as the WB-ACAT model captured seven distinct sIEC segments, the flux values for each segment were set as upper bound on the one sIEC model corresponding to this segment. Step 5 (COBRA modeling): for each sIEC model, FBA was performed with the levodopa luminal uptake reaction or basolateral secretion as objective function, depending on the mode. Step 6 (COBRA modeling): the obtained FBA flux values for each sIEC model were set as parameters for the derivatives in the corresponding sIEC segments of the WB-ACAT model. The new rates initialized the next time step in the PBPK modeling (Step 3). Numbers with single and double subscript correspond to competition and trans-stimulation, respectively. COBRA, constraint-based reconstruction and analysis; PBPK, physiologically based pharmacokinetic; sIEC, small-intestine epithelial cell; WB-ACAT, whole-body advanced compartmental absorption and transit.

PRD is based on the redistribution of the daily protein allowance to the last meal, for the latest stages of PD.^8^ It has been demonstrated that gastric emptying rate (GER) decreases severely in the latest stage of PD. Moreover, the kinetics of amino acids in the plasma, after protein ingestion, are higher than the baseline after 8 h.^23^ We consequently assumed that a high fraction of amino acids is present in the systemic circulation and in the portal vein, particularly, when the next levodopa dose is taken the following day. In particular, the trans-stimulation effect on levodopa secretion by amino acids was captured with the sIEC basolateral transporters (Figure 4). The simulation showed that a higher flux through the basolateral antiporter induced a higher bioavailability of levodopa, which is reflected by the increase of AUC above the efficacy threshold throughout the day (11.23%) in comparison to the fasted state (Figure 3c). Taken together, these results support the observed superiority of PRD over LPD to increase the systemic bioavailability of levodopa, which is reflected in our simulations by the cumulative increase in the AUC (34%).

### Impaired gastrointestinal processes reduced levodopa efficacy

The slower gastric motility, induced by food and several conditions, decreases the bioavailability of orally absorbed drugs,^7^ including levodopa. We identified the parameter for GER for the fasted and the fed state based on a two-occasion study^7^ (see Methods for details, Table 1). We used the fitted GER to simulate the kinetics of levodopa in PD patients with the concomitant administration of food (Figure 3a). The parameter sensitivity analysis with respect to levodopa plasma concentrations showed that the top 5 ranking parameters among the 243 whole-body parameters were gastrointestinal parameters (Supplementary Figure S4, Supplementary Table S4). These findings highlight the importance of oral absorption in the dynamics of levodopa and consequently the emergence of fluctuations in the motor response.

### Ranking of amino acids effects toward levodopa bioavailability revealed serine-rich diet as a potential augmenting diet for levodopa *in silico*

**Figure 5:**
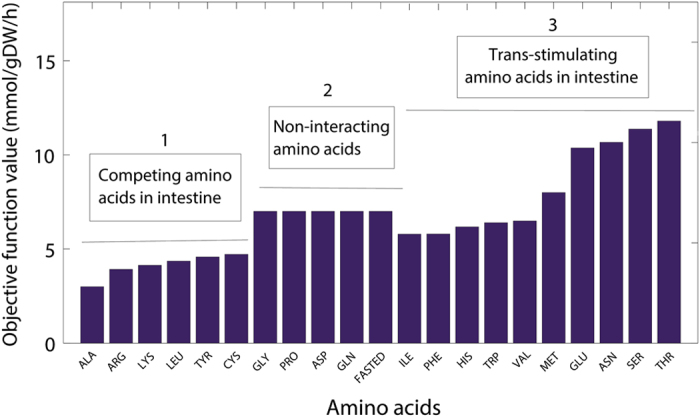
Ranking of amino acids with respect to the levodopa fraction that reaches the brain. Amino acids with lowest ranking were those which competed with levodopa in the intestine. A second set of amino acids did not interact with levodopa and, consequently, the levodopa pharmacokinetics was identical to those of the fasted state. A third set improved the levodopa absorption and also did not compete with amino acid uptake by the brain. The objective function in the simulations was the levodopa transport reaction across the blood–brain barrier using an extended sIEC model, to which a kidney and brain compartment with the corresponding levodopa transport reactions were added. sIEC, small-intestine epithelial cell.

The competition and trans-stimulation between levodopa and amino acids take place mainly in the gut, brain, and kidneys.^24^ To capture also these interactions in our WB-ACAT-sIEC model, we extended the sIEC model by a kidney and brain compartment (Supplementary Table S5) and included the corresponding levodopa transport reactions (sIEC*, supplementary section amino acids ranking simulations). To simulate this interaction between the three organs in the levodopa bioavailability, we set the levodopa influx rate to the intestinal lumen to the arbitrary value of 15 mmol/g of dry weight/h (Supplementary Table S6), of which 66% could reach the systemic circulation in the fasting state and 30% of the absorbed fraction was eliminated by the kidneys, in accordance with experimental data.^7^ We then simulated the simultaneous administration of levodopa with the different amino acids by selecting as objective function the brain transport reaction for levodopa. This simulation allowed us to systematically determine, which amino acid would lead to a higher flux of levodopa transported to the brain and thus, could result in a better clinical outcome. We found that threonine, serine, and asparagine resulted in the highest brain bioavailability of levodopa (Figure 5). To our knowledge, these amino acids have not been reported to compete with levodopa in the small intestine and in the brain. Moreover, the amino acids were predicted to compete with levodopa for elimination in the kidneys and trans-stimulate levodopa secretion from the intestinal lumen. It has been shown that serine improves dopamine production.^25^ Consequently, we ranked serine as the amino acid with the highest contribution to levodopa bioavailability.

Using the WB-ACAT-sIEC model, we predicted that the addition of serine in the systemic circulation could improve the bioavailability of levodopa as shown by the increase of the AUC above the efficacy threshold (22.02%) (Figure 3d,e). The subsequent increase of amino acids concentration in the plasma improved the bioavailability of the next dose through a higher absorption in the basolateral side of the seven compartments of the small intestine. Taken together, we propose that a serine-rich meal after a levodopa dose could improve the brain bioavailability of levodopa.

## Discussion

Motivated by the observation that protein-containing meals can alter the levodopa bioavailability and thus the motor symptoms of PD patients, we developed a multiscale PBPK–COBRA model of the gastrointestinal tract. We then investigated different dietary strategies. Our key results include: (i) late-stage, levodopa-treated PD patients would benefit the most by dietary intervention; (ii) PRD but not LPD improved the bioavailability of levodopa; (iii) gastrointestinal transit and loss of levodopa explained most of the variability in levodopa bioavailability; and (iv) serine-rich diet could increase the brain levodopa bioavailability. Taken together, we demonstrate that computational modeling could add further mechanistic insight into the diet-levodopa interactions and may be used to propose PD patient-specific dietary intervention strategies.

### The combined model: construction, assumptions and validation

In this study, we developed a spatially, temporally, and mechanistically detailed model of the human gastrointestinal tract (Figure 1) by combining two powerful modeling techniques: PBPK and COBRA. In comparison to the other efforts,^26^ we demonstrate here that this hybrid modeling technique can be further expanded by including more refined PBPK models (i.e., the ACAT model), as well as by integrating more than one stoichiometric metabolic models (i.e., seven sIEC models) (Figure 1). Importantly, the simulation settings were consistent with literature reports, such as levodopa being absorbed equally in all the parts across small intestinal,^21^ while amino acids were only absorbed in the proximal jejunum (jejunum 1 and 2 in the model).^27^ The kinetic profile of levodopa with concomitant administration of proteic diet in late-stage patients (Figure 3a) matched the profile of levodopa of one case patient with gastrointestinal resection,^28^ which suggests that inhibitory amino acids completely block the access of levodopa to the intestinal transporters in the sites of absorption. These findings show that the proposed hybrid modeling approach (Figure 1) provides a powerful tool to assess diet–drug interactions, which requires interrogation at the physiological, as well as biochemical level.^29^

### Late-stage, levodopa-treated PD patients would benefit the most by dietary intervention

In the last stages of PD, levodopa-treated patients experience fluctuations in the motor response.^30^ The on-off phenomena are correlated to inadequate concentrations of levodopa reaching the brain. Since the impairment of the gastrointestinal motility in PD^31^ increases with disease progression, the erratic absorption of levodopa, especially with diet, is one of the factors causing motor fluctuations. As our model showed (Figure 3a), the decrease in levodopa absorption was lower with respect to the therapeutic threshold in early-stage patients with low impairment of gastric emptying, while it substantially decreased the bioavailability of levodopa in later stages of PD. These results are in agreement with reported clinical trials,^30^ and routine, where HY 3 and HY 4 patients are recommended to follow a dietary plan, such as PRD and LPD.

### Gastrointestinal transit and loss of levodopa explained most of the variability in levodopa bioavailability

Constipation is a clinical symptom associated with PD, particularly at the later stages.^31^ Overall, the GER is inversely proportional to the disease stage.^32^ In addition, levodopa is degraded in the stomach and intestine lumen as a consequence of gut microbiota,^33^ luminal enzymes,^5^ and chemical degradation.^11^ The higher residence time of levodopa in the gastrointestinal tract, caused by the slower GER, leads to a higher degraded fraction. The predicted decrease in the maximal concentration (*C*_max_) was the result of the combination of a slow GER and the luminal degradation (Figure 3a). The parameter sensitivity analysis showed that gastric and intestinal processes were the most influential factors for levodopa bioavailability (Supplementary Table S4, Supplementary Figure S4). The GER has been shown to be the main parameter that induces a delay in levodopa absorption.^32^ Our observation is further consistent with the reported decrease in levodopa efficiency with pH^34^ and *Helicobacter pylori* infection.^35,36^ The levodopa loss in the stomach also motivated approaches that bypassed the gastrointestinal tract^37^ and the intestine,^5^ as well as provides a rationale for investigational prokinetics for PD patients.^38^

The absorptive profile of levodopa has been reported to show multiple peaks in plasma and an erratic absorption. Such erratic kinetics were not observed in the simulations (Figure 3a), which suggests that GER is a time-dependent parameter, as suggested previously.^39^ Our results suggest that the plasmatic concentrations of levodopa were higher 2 h after the administration than the a.c. administration. This observation could be explained by the absorption of levodopa in the ileum, where the competition with amino acids has not been reported.^27^ This finding indicates a role of ileal levodopa absorption in the formation of delayed plasmatic peaks.

### PRD but not LPD improved the bioavailability of levodopa

It has been shown that PRD had a better clinical outcome than LPD.^8^ *In silico,* the PRD had also a better performance due to the improved bioavailability of levodopa (Figure 3b,c). A combination of factors has been suggested to result in the superiority of protein redistribution diet.^8^ In LPD, we showed that competing amino acids decreased the levodopa peak (Figure 3a,b). It is likely that the decrease is more pronounced with impaired GER. A slower GER potentializes the loss of levodopa by competition through exposing the dietary proteins to intestinal peptidases for longer periods of time, thus releasing amino acids. It has been shown that in healthy volunteers after protein intake, a minor part of the diet is transformed into free amino acids in the small intestine, while the major part forms di- and tri-peptides and is absorbed by PEPT1.^40^ A clinical trial conducted on healthy volunteers showed no difference in the pharmacokinetics of levodopa when absorbed alone or with a solution of proteins, which questioned the influence of the gastrointestinal processes on the absorption of levodopa.^41^ With most proteins being transformed into non-competing peptides, levodopa is not subjected to competition for luminal transporters. Thus, the delay in GER exacerbates the competitive potential of dietary amino acids, which leads to higher loss of levodopa in the small intestine.

### Serine-rich diet could increase the brain levodopa bioavailability

Basolateral amino acids, mimicking the post prandial state, have been shown to trans-stimulate the secretion of levodopa.^9^ The latter finding provides opportunities for augmenting dietary intervention. Serine supplementation increased *in silico* the bioavailability of levodopa for PD patients with moderately impaired GER (Figure 3d). Furthermore, the exchange of levodopa with amino acids could be a clearance route for amino acids, thus, preventing further competition in the brain (Supplementary Figure S5). Serine also modulates the activation of *N*-methyl-d-aspartate class of glutamate receptors (NMDARs), which were shown to be involved in dopamine synthesis and release.^25^ Thus, serine-rich diet could improve the absorption of levodopa and the production of dopamine.^42^ Recently, a clinical study on late-stage PD patients has demonstrated a higher bioavailability of levodopa and an improvement of ‘on’ times with soybean.^43^ Given that soybean is mainly composed of 2331 mg/100 g of glutamate, 1411 mg/100 g of aspartate, 982 mg/100 g of arginine, and 687 mg/100 g of serine, it appears that inhibitory effects of arginine are counteracted by the predicted beneficial effects of serine and glutamate, while aspartate was predicted to be neutral (Figure 5). This finding suggests that a cumulative, dose-dependent effect of amino acids on the ranking scale that we have developed (Figure 5), in a given diet, is a good assessment of its effects on the pharmacokinetics of levodopa. A higher bioavailability of levodopa allows (i) a better clinical outcome, (ii) a decrease in the daily dose with (iii) the subsequent decrease in adverse reactions.

Taken together, we demonstrate in this study that the combination of genome scale and dynamical models can be used to assess the diet–drug interactions and can provide a valuable tool to design nutritional intervention strategies.

## Materials and methods

To generate the multiscale gastrointestinal model, we (i) expanded the whole-body generic PBPK model^44^ by the ACAT model,^11^ (ii) added levodopa transport reactions to the sIEC model,^9^ and (iii) coupled both models with respect to a specific time step.

### Whole-body PBPK modeling and system identification

The ACAT model^11^ and the whole-body generic PBPK (Figure 1, Supplementary Figure S1A)^10^ model were implemented and combined in Matlab (2014b release, MathWorks, Natick, MA, USA). Details on the models and their integration can be found in the supplementary section dynamical modeling of levodopa. The whole-body generic PBPK model with ACAT component consisted of 42 ordinary differential equations and 243 parameters. In the following, we will refer to this ACAT-expanded whole-body generic model as WB-ACAT model.

The kinetic parameters of levodopa (Supplementary Tables S1 and S2) were identified through fitting of the WB-ACAT model on averaged data from 24 fasting healthy volunteers' plasma concentrations after oral (per os) administration of 200 mg of a standard formulation of levodopa and 50 mg of peripheral metabolism inhibitor (benserazide)^45^ (Supplementary section parameter estimation; Supplementary Figure S1B). The goodness of fit was assessed through visual inspection (Supplementary Figure S2), the Kolmogorov–Smirnov test^46^ with a resulting *P*=0.9994, and the Pearson’s correlation coefficient with r=0.99 (*P*<0.0001).

The delay in the GER parameter induced by food was inferred by sequentially fitting data from a two-occasion study:^7^ plasma levodopa concentrations in the fasted state and plasma levodopa concentrations after aproteic meal. For the first occasion (fasted), all model parameters were estimated except GER, which was set, as reported in the literature, for the fasting state (Table 1). For the second occasion (fed), all kinetic parameters were fixed and only GER was estimated. The sequential fitting approach was undertaken, as it allowed quantification of varying parameter between fed and fasted states.

The time-dependent parameter sensitivity analysis with respect to levodopa concentrations in plasma was computed as the result of the following non-normalized time-dependent derivative:

$$\mathrm{parameter}\mathrm{sensitivity}=\frac{\partial y}{\partial x}$$

where *x* represents the vector of all WB-ACAT model parameters and *y* represents the plasma concentrations of levodopa. Then, the absolute time integral for every parameter was computed for every parameter using *trapz* function in Matlab.

### sIEC model

To take into account the gut wall metabolism and transport, the sIEC stoichiometric model was used. The sIEC model contains a thorough collection of metabolic and transport pathways of amino acids, which provided nonintuitive metabolite utilization strategies in different conditions.^16^ To account for levodopa uptake and secretion by the transporters (EntrezGene ID: 11067-6591, 117247, 23428-6520), 36 levodopa reactions were added to the model, representing the specific stoichiometric coefficients for the transported co-metabolites accounting for competition and trans-stimulation (supplementary section adding levodopa reactions to sIEC) in the reported order of affinity^24^ as measured experimentally.^9^ The resulting sIEC_levodopa model, to which we will refer in the remainder as sIEC model, consisted of 433 metabolites, 1318 reactions, and 616 genes.

### Coupling algorithm

The coupling between seven sIEC models and the WB-ACAT model (Figures 1 and 4) was achieved by performing dynamic flux balance analysis,^47^ which computes uptake rates of each of the seven sIEC models using as constraints the outcome of the simulation of the WB-ACAT model, which were subsequently set as derivatives $\left(\frac{dX}{dt}\right)$ in the WB-ACAT model. We implemented this coupling with the static optimization approach (SOA),^48^ which discretizes the simulation time into a defined number of steps and simulates sequentially the PBPK and COBRA model for every step^48^ (Figure 4). The total simulation time of 18 h was divided into steps of 0.1 h, which represents a good compromise between the integration tolerance of the dynamical model, the simulation time, and the steady state assumption of the sIEC model with respect to the fast kinetics of levodopa in the blood following per os administration.^49^ SOA assumes steady state of the metabolic network in intervals of 0.1 h, the sIEC model will reach a different steady state depending on the changing constraints of levodopa between the different time intervals.

## Figures and Tables

**Table 1:** Fed versus fasted state physiological parameters

*Parameters*	*Fasted/ante cibum*	*Fed*
GER	3.96 per h	0.33 per h
Stomach volume	50 ml	1,000 ml
Colon volume	1,000 ml	7,000 ml
Small intestine transit rate	2.1 per h	0.57 per h
Gastric pH	2	5

Abbreviation: GER, gastric-emptying rate.

The physiological parameters in fasted and fed state were taken from the literature.^49^ The gastric-emptying rate was estimated by sequential fit on levodopa plasma concentrations in fasted and fed states onto the WB-ACAT model.

## References

[bib1] Lennernas, H. Human intestinal permeability. J. Pharm. Sci. 87, 403–410 (1998). 9548891 10.1021/js970332a

[bib2] Brooks, D. J. Optimizing levodopa therapy for Parkinson's disease with levodopa/carbidopa/entacapone: implications from a clinical and patient perspective. Neuropsychiatr. Dis. Treat. 4, 39–47 (2008). 18728816 10.2147/ndt.s1660 PMC2515910

[bib3] Eisenhofer, G. et al. Substantial production of dopamine in the human gastrointestinal tract. J. Clin. Endocrinol. Metab. 82, 3864–3871 (1997). 9360553 10.1210/jcem.82.11.4339

[bib4] Fahn, S. & Poewe, W. Levodopa: 50 years of a revolutionary drug for Parkinson disease. Mov. Disord. 30, 1–3 (2015). 25488146 10.1002/mds.26122

[bib5] Nutt, J. G., Woodward, W. R., Hammerstad, J. P., Carter, J. H. & Anderson, J. L. The ‘on-off’ phenomenon in Parkinson's disease. Relation to levodopa absorption and transport. N. Engl. J. Med. 310, 483–488 (1984). 6694694 10.1056/NEJM198402233100802

[bib6] Ishihara, L. & Brayne, C. A systematic review of nutritional risk factors of Parkinson's disease. Nutr. Res. Rev. 18, 259–282 (2005). 19079910 10.1079/NRR2005108

[bib7] Contin, M. & Martinelli, P. Pharmacokinetics of levodopa. J. Neurol. 257, S253–S261 (2010). 21080186 10.1007/s00415-010-5728-8

[bib8] Cereda, E., Barichella, M., Pedrolli, C. & Pezzoli, G. Low-protein and protein-redistribution diets for Parkinson's disease patients with motor fluctuations: a systematic review. Mov. Disord. 25, 2021–2034 (2010). 20669318 10.1002/mds.23226

[bib9] Camargo, S. M. et al. The molecular mechanism of intestinal levodopa absorption and its possible implications for the treatment of Parkinson's disease. J. Pharmacol. Exp. Ther. 351, 114–123 (2014). 25073474 10.1124/jpet.114.216317

[bib10] Jones, H. & Rowland-Yeo, K. Basic concepts in physiologically based pharmacokinetic modeling in drug discovery and development. CPT Pharmacometrics Syst. Pharmacol. 2, e63 (2013). 23945604 10.1038/psp.2013.41 PMC3828005

[bib11] Agoram, B., Woltosz, W. S. & Bolger, M. B. Predicting the impact of physiological and biochemical processes on oral drug bioavailability. Adv. Drug Deliv. Rev. 50 Suppl 1, S41–S67 (2001). 11576695 10.1016/s0169-409x(01)00179-x

[bib12] O'Brien, E. J., Monk, J. M. & Palsson, B. O. Using genome-scale models to predict biological capabilities. Cell 161, 971–987 (2015). 26000478 10.1016/j.cell.2015.05.019 PMC4451052

[bib13] Schellenberger, J. et al. Quantitative prediction of cellular metabolism with constraint-based models: the COBRA Toolbox v2.0. Nat. Protoc. 6, 1290–1307 (2011). 21886097 10.1038/nprot.2011.308 PMC3319681

[bib14] Thiele, I. & Palsson, B. O. A protocol for generating a high-quality genome-scale metabolic reconstruction. Nat. Protoc. 5, 93–121 (2010). 20057383 10.1038/nprot.2009.203 PMC3125167

[bib15] Beard, D. A., Liang, S. D. & Qian, H. Energy balance for analysis of complex metabolic networks. Biophys. J. 83, 79–86 (2002). 12080101 10.1016/S0006-3495(02)75150-3 PMC1302128

[bib16] Sahoo, S. & Thiele, I. Predicting the impact of diet and enzymopathies on human small intestinal epithelial cells. Hum. Mol. Genet. 22, 2705–2722 (2013). 23492669 10.1093/hmg/ddt119 PMC3674809

[bib17] Duarte, N. C. et al. Global reconstruction of the human metabolic network based on genomic and bibliomic data. *Proc. Natl Acad. Sci. USA* 104, 1777–1782 (2007). 17267599 10.1073/pnas.0610772104 PMC1794290

[bib18] Thiele, I. et al. A community-driven global reconstruction of human metabolism. Nat. Biotechnol. 31, 419–425 (2013). 23455439 10.1038/nbt.2488 PMC3856361

[bib19] Sahoo, S., Aurich, M. K., Jonsson, J. J. & Thiele, I. Membrane transporters in a human genome-scale metabolic knowledgebase and their implications for disease. Front. Physiol. 5, 91 (2014). 24653705 10.3389/fphys.2014.00091 PMC3949408

[bib20] Sahoo, S., Haraldsdottir, H. S., Fleming, R. M. & Thiele, I. Modeling the effects of commonly used drugs on human metabolism. FEBS J. 282, 297–317 (2015). 25345908 10.1111/febs.13128

[bib21] Lennernas, H. et al. The effect of L-leucine on the absorption of levodopa, studied by regional jejunal perfusion in man. Br. J. Clin. Pharmacol. 35, 243–250 (1993). 8471400 10.1111/j.1365-2125.1993.tb05691.x PMC1381569

[bib22] Hardoff, R. et al. Gastric emptying time and gastric motility in patients with Parkinson's disease. Mov. Disord. 16, 1041–1047 (2001). 11748735 10.1002/mds.1203

[bib23] Bos, C. et al. Postprandial kinetics of dietary amino acids are the main determinant of their metabolism after soy or milk protein ingestion in humans. J. Nutr. 133, 1308–1315 (2003). 12730415 10.1093/jn/133.5.1308

[bib24] Verrey, F., Meier, C., Rossier, G. & Kuhn, L. C. Glycoprotein-associated amino acid exchangers: broadening the range of transport specificity. Pflugers Arch. 440, 503–512 (2000). 10958334 10.1007/s004240000274

[bib25] Gelfin, E. et al. D-serine adjuvant treatment alleviates behavioural and motor symptoms in Parkinson's disease. Int. J. Neuropsychopharmacol. 15, 543–549 (2012). 21733283 10.1017/S1461145711001015

[bib26] Krauss, M. et al. Integrating cellular metabolism into a multiscale whole-body model. PLoS Comput. Biol. 8, e1002750 (2012). 23133351 10.1371/journal.pcbi.1002750 PMC3486908

[bib27] Adibi, S. A., Gray, S. J. & Menden, E. The kinetics of amino acid absorption and alteration of plasma composition of free amino acids after intestinal perfusion of amino acid mixtures. Am. J. Clin. Nutr. 20, 24–33 (1967). 6017006 10.1093/ajcn/20.1.24

[bib28] Nagayama, H. et al. Pharmacokinetics of levodopa before and after gastrointestinal resection in parkinson's disease. Case Rep. Neurol. 7, 181–185 (2015). 26500544 10.1159/000381181 PMC4608656

[bib29] van der Graaf, P. H. & Benson, N. Systems pharmacology: bridging systems biology and pharmacokinetics-pharmacodynamics (PKPD) in drug discovery and development. Pharm. Res. 28, 1460–1464 (2011). 21560018 10.1007/s11095-011-0467-9

[bib30] Contin, M. et al. Levodopa therapy monitoring in patients with Parkinson disease: a kinetic-dynamic approach. Ther. Drug Monit. 23, 621–629 (2001). 11802094 10.1097/00007691-200112000-00005

[bib31] Fasano, A., Visanji, N. P., Liu, L. W., Lang, A. E. & Pfeiffer, R. F. Gastrointestinal dysfunction in Parkinson's disease. Lancet Neurol. 14, 625–639 (2015). 25987282 10.1016/S1474-4422(15)00007-1

[bib32] Doi, H. et al. Plasma levodopa peak delay and impaired gastric emptying in Parkinson's disease. J. Neurol. Sci. 319, 86–88 (2012). 22632782 10.1016/j.jns.2012.05.010

[bib33] Sousa, T. et al. The gastrointestinal microbiota as a site for the biotransformation of drugs. Int. J. Pharm. 363, 1–25 (2008). 18682282 10.1016/j.ijpharm.2008.07.009

[bib34] Rivera-Calimlim, L., Dujovne, C. A., Morgan, J. P., Lasagna, L. & Bianchine, J. R. L-dopa treatment failure: explanation and correction. Br. Med. J. 4, 93–94 (1970). 5482325 10.1136/bmj.4.5727.93 PMC1819624

[bib35] Hashim, H. et al. Eradication of Helicobacter pylori infection improves levodopa action, clinical symptoms and quality of life in patients with Parkinson's disease. PLoS ONE 9, e112330 (2014). 25411976 10.1371/journal.pone.0112330 PMC4239049

[bib36] Pierantozzi, M. et al. Helicobacter pylori eradication and l-dopa absorption in patients with PD and motor fluctuations. Neurology 66, 1824–1829 (2006). 16801644 10.1212/01.wnl.0000221672.01272.ba

[bib37] Foltynie, T. et al. Impact of Duodopa on quality of life in advanced Parkinson's disease: a UK case series. Parkinsons Dis. 2013, 362908 (2013). 23476888 10.1155/2013/362908 PMC3586488

[bib38] Sanger, G. J. et al. GSK962040: a small molecule, selective motilin receptor agonist, effective as a stimulant of human and rabbit gastrointestinal motility. Neurogastroenterol. Motil. 21, 657–664, e30-1 (2009). 19374732 10.1111/j.1365-2982.2008.01270.x

[bib39] Waller, D. G., Usman, F., Renwick, A. G., Macklin, B. & George, C. F. Oral amino acids and gastric emptying: an investigation of the mechanism of levodopa-induced gastric stasis. Br. J. Clin. Pharmacol. 32, 771–773 (1991). 1768574 PMC1368563

[bib40] Adibi, S. A. & Mercer, D. W. Protein digestion in human intestine as reflected in luminal, mucosal, and plasma amino acid concentrations after meals. J. Clin. Invest. 52, 1586–1594 (1973). 4718954 10.1172/JCI107335 PMC302429

[bib41] Robertson, D. R. et al. The influence of protein containing meals on the pharmacokinetics of levodopa in healthy volunteers. Br. J. Clin. Pharmacol. 31, 413–417 (1991). 2049250 10.1111/j.1365-2125.1991.tb05555.x PMC1368327

[bib42] Growdon, J. H., Melamed, E., Logue, M., Hefti, F. & Wurtman, R. J. Effects of oral L-tyrosine administration on CSF tyrosine and homovanillic acid levels in patients with Parkinson's disease. Life Sci. 30, 827–832 (1982). 6175872 10.1016/0024-3205(82)90596-3

[bib43] Nagashima, Y., Kondo, T., Sakata, M., Koh, J. & Ito, H. Effects of soybean ingestion on pharmacokinetics of levodopa and motor symptoms of Parkinson's disease—In relation to the effects of Mucuna pruriens. J. Neurol. Sci. 361, 229–234 (2016). 26810548 10.1016/j.jns.2016.01.005

[bib44] Peters, S. A. Evaluation of a generic physiologically based pharmacokinetic model for lineshape analysis. Clin. Pharmacokinet. 47, 261–275 (2008). 18336055 10.2165/00003088-200847040-00004

[bib45] Keller, G. A. et al. Comparative bioavailability of 2 tablet formulations of levodopa/benserazide in healthy, fasting volunteers: a single-dose, randomized-sequence, open-label crossover study. Clin. Ther. 33, 500–510 (2011). 21635995 10.1016/j.clinthera.2011.04.012

[bib46] Massey, F. J. The Kolmogorov-Smirnov test for goodness of fit. J. Am. Stat. Assoc. 46, 68–78 (1951).

[bib47] Varma, A. & Palsson, B. O. Stoichiometric flux balance models quantitatively predict growth and metabolic by-product secretion in wild-type Escherichia coli W3110. Appl. Environ. Microbiol. 60, 3724–3731 (1994). 7986045 10.1128/aem.60.10.3724-3731.1994 PMC201879

[bib48] Mahadevan, R., Edwards, J. S. & Doyle, F. J. 3rd Dynamic flux balance analysis of diauxic growth in Escherichia coli. Biophys. J. 83, 1331–1340 (2002). 12202358 10.1016/S0006-3495(02)73903-9 PMC1302231

[bib49] Covert, M. W., Xiao, N., Chen, T. J. & Karr, J. R. Integrating metabolic, transcriptional regulatory and signal transduction models in Escherichia coli. Bioinformatics 24, 2044–2050 (2008). 18621757 10.1093/bioinformatics/btn352 PMC6702764

