# Supplementary material for: Model-based dietary optimization for late-stage, levodopa-treated, Parkinson’s disease patients
Source: NPJ Syst Biol Appl. 2016 Jun 16;2:16013–. doi: 10.1038/npjsba.2016.13 (PMC5516849; doi:10.1038/npjsba.2016.13)
Supplement: Supplementary Information [file npjsba201613-s1.doc]

**Supplementary text:**

**Model-based dietary optimization for late-stage, levodopa-treated, Parkinson’s disease patients.**

**Marouen Ben Guebila and Ines Thiele**

**Dynamical modeling of levodopa gastrointestinal transit and metabolism within the whole body generic PBPK model**

The generic physiologically based pharmacokinetic whole body model (1) assumes the enterocyte as a ‘thin wall’. In other words, drugs absorbed in the lumen are directly present in the portal vein. This assumption does not allow us to model luminal competition and basolateral trans-stimulation. For these reasons, enterocyte compartments were added as described in (2) that aided in the coupling of sIEC genome-scale metabolic models. The enterocyte performs three main biological functions with regards to levodopa. The luminal absorption, the basolateral secretion and the accumulation of levodopa which is the difference between the absorption and secretion.

The absorption and secretion by the enterocyte was modeled with the following ODEs (2):

Luminal absorption in compartment
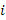
:


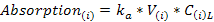


Basolateral secretion in compartment
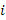
:


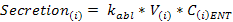


Concentration inside the enterocyte in compartment
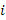
:


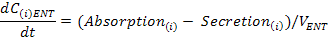


with
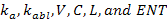
 luminal absorption constant, basolateral absorption constant, volume, concentration, lumen, enterocyte, respectively. *i* ranges from 1 to 7.

The set of equations and parameters describing gastrointestinal processes were added to the generic PBPK whole body model as described in (1), as in table S1.

**Parameter estimation**

The data fitting and simulations was performed using Matlab (2014b release, MathWorks, Natick, MA) and Tomlab (TomOpt Inc.). The model parameters (Table S1) were identified with fmincon algorithm, which finds the minimum of a nonlinear optimization problem subjected to a set of constraints, and global search option, that ensures the converges to the global minimum of the objective function through testing different sets of initial points. The parameters were constrained to stay in the reported human biological values (Table S1). The minimized objective function is chosen as the root mean square deviation (RMSD).

**Adding levodopa reactions to sIEC**

The sIEC model is represented in the stoichiometric matrix S, which is obtained by the conversion of the reconstruction into a mathematical format as following:

Reaction:
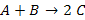
 (1)


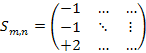
 (2)

where the rows represent metabolites and the columns represent reactions.

The levodopa transport reactions were added to the sIEC model (Table S2) and the luminal uptake of levodopa was set as the objective function. The choice of the objective function agreed with both empirical results and numerical simulations. The main function of the small intestine in the gut wall is the transport and metabolism of substrates from the luminal side to the portal vein. Since levodopa does not contribute to the homeostasis of the sIEC, considering biomass maintenance as the objective function did not result in transport of levodopa. The luminal and basolateral transport reactions were added according to the recently identified levodopa transporters (3).

The competition was modeled as the loss of a luminal fraction of levodopa in the presence of amino acids. Stoichiometric coefficients were calibrated to account for affinities of different amino acids for the antiporter (Table S2 – luminal competition). In fact, amino acids with high affinity to the transporter (cystine) result in higher loss of levodopa, thus they were assigned a higher coefficient than less affine amino acids (ornithine) for the transporter. The coefficients for leucine and arginine were measured *in vivo* in mice (3). While the unmeasured values were extrapolated following the reported order of affinity (4).

The trans-stimulation was modeled as the decrease in the flux through cellular accumulation reaction (Table S2 – Intracellular accumulation), by unconstraining the basolateral antiporter with respect to the fasted value, and by setting the flux of the basolateral uniporter to the fasted value. The resulting basolateral transport was then updated and set in the ODE model as the gastrointestinal basolateral efflux term in the next time step. The percentage decrease in the intracellular accumulation of levodopa was set to 0.7 as measured *in vivo* in mice (3) (Table S2 – Trans-stimulation). It can be adapted to account for different basolateral affinities of amino acids. In fact, basolateral amino acids that trigger a high trans-stimulation through the antiporter reduce the intracellular accumulation of levodopa by a higher coefficient than less trans-stimulating amino acids. Consequently, the new flux through the antiporter was computed with the following linear programming problem:


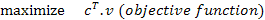


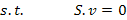


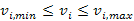


where
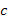
 is the coefficients vector for the objective function,
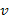
 represents the flux distribution,
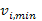
and
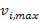
 are respectively the minimal and maximal capacity for reaction
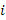
 .The system assumes steady state for the chosen time step as well as mass balance (
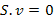
). This approach is also called flux balance analysis (FBA) (5).

**Conversion from mg/hour to mmol/gDW_enterocyte_/hour**

The conversion from
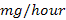
 to
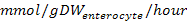
 was done as follows:
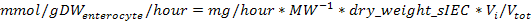
, with
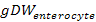
, the dry weight of enterocytes in gram,
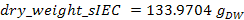
 the dry weight of the small intestine enterocyte,
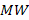
 the molecular weight of levodopa,
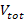
 the total volume of the small intestine , and
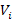
 the volume of compartment
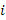
, with
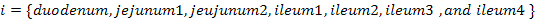
.

**Sequential fit on two occasions: fasted and fed states**

First, the fitting of whole body generic PBPK model on fasted healthy volunteers plasma concentrations of levodopa, after 200 mg of standard formulation of levodopa, allowed to get the distribution and elimination parameters including the GER at the fasted state (3.96 h^-1^). Then, all model parameters were fixed and only GER was estimated (0.33 h^-1^) in the second occasion, i.e., the administration of levodopa with aproteic meal.

## Amino acids ranking simulations

The small intestine model was extended by adding a kidney compartment and brain compartment with the corresponding levodopa transport reactions (Figure S5). The objective function was set as the demand reaction for levodopa in the brain (Table S4). The kidney and brain exchange reaction modeling took into account the affinity of substrates for the transporter, through assigning different arbitrary weights (Table S2) for the fluxes representing each individual amino acid (Table S5). The weighing coefficient is proportional to the amount of levodopa lost due to the competition with amino acids. The higher the competitive potential of the amino acid, the higher the assigned weight following the reported order of affinity of each transporter for amino acids (4). The coefficients were calibrated to take into account the tissue-specific competition potential of levodopa transporters in the following order: blood brain barrier, enterocyte, kidneys as supported by clinical investigation (6, 7).Trans-stimulation was not modeled in the kidneys and the brain as it is not supported by experimental evidence. The liver elimination was not formulated in the optimization problem as no records of competition were reported.

An arbitrary intake of levodopa was set to 15 mg/l/h. Two thirds of this amount are absorbed by the small intestine. Since levodopa is antiported to the blood compartment, an excess of alanine, the contralateral substance to levodopa, was added. In order for levodopa to reach the blood brain barrier and the kidney, two demand reactions were added in these compartments. 30% of levodopa was set to be eliminated by the kidneys and the rest transported through the blood brain barrier. Then a 3 mg/l/h of each amino acid was added simultaneously with levodopa and the fraction reaching the brain was calculated. Each amino acid either competed with levodopa in the small intestine, blood brain barrier or kidneys or all of the three organs. Basolateral trans-stimulation of levodopa intestinal secretion increases the absorption of levodopa. Since trans-stimulation was not proven in kidney and brain transporters, these were not modeled.

**Figures:**

**
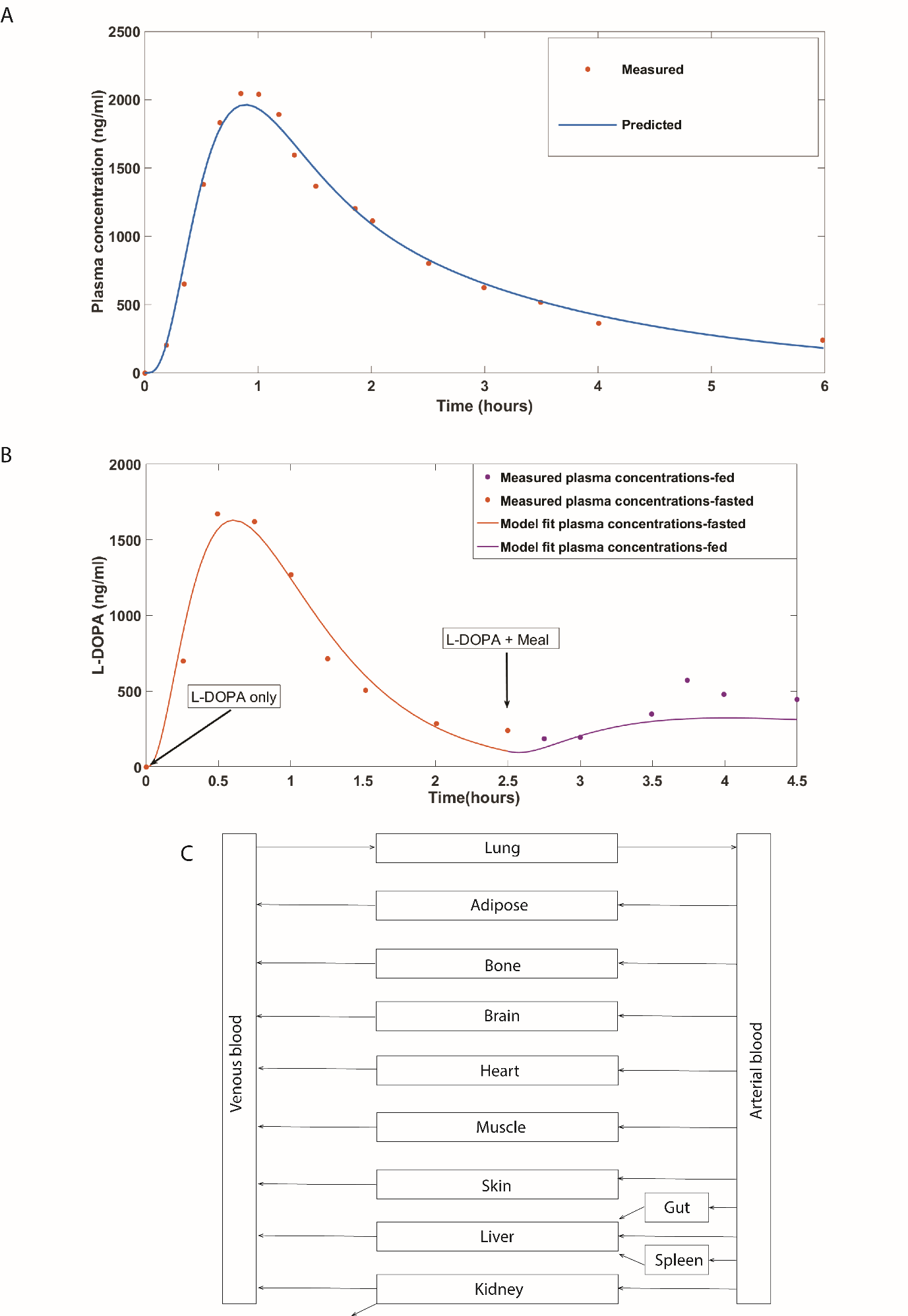
**

**Figure S1** – **A** – levodopa PBPK whole body model structure. The small intestine compartmentalization is detailed in Figure 3. The elimination is considered only in the kidneys, while peripheral metabolism of levodopa (e.g., liver) as well as conversion to dopamine (in the brain) represent routes of elimination. The goodness of fit of the latter models on the same data did not show improvement over the kidney elimination model (data not shown). **B** - Curve fitting of a standard dose of 200 mg of carbidopa (levodopa and benserazide) in healthy volunteers on PBPK generic whole body model. The solid lines represent the model performance with the estimated parameters with the observed data values. The root mean square deviation value was 71.13 ng/ml. **C** - Two occasions sequential fit of levodopa plasma concentrations in fasted state followed by a meal (8). The parameters identified in the fasted state were fixed in fed state except the gastric emptying rate, which was left unconstrained and was estimated in the second administration of levodopa with a meal. The solid line represent the model simulation with the parameters obtained from the fitting process.


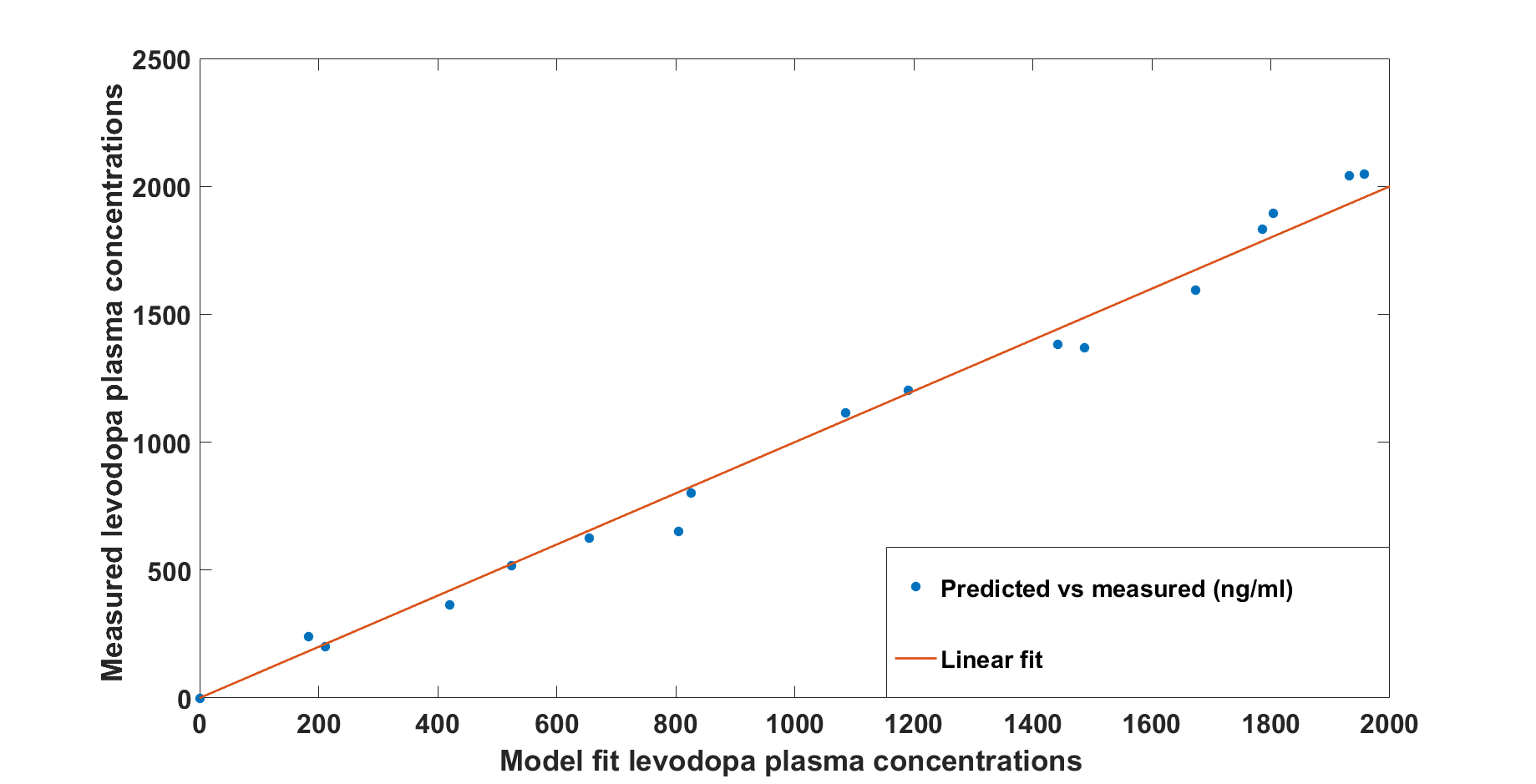


**Figure S2** – Goodness of fit plot of the simulated and measured levodopa plasma concentration in the fasted state, corresponding to figure S1-B. The model was simulated with the parameter values obtained from curve fitting the model on the observed data.


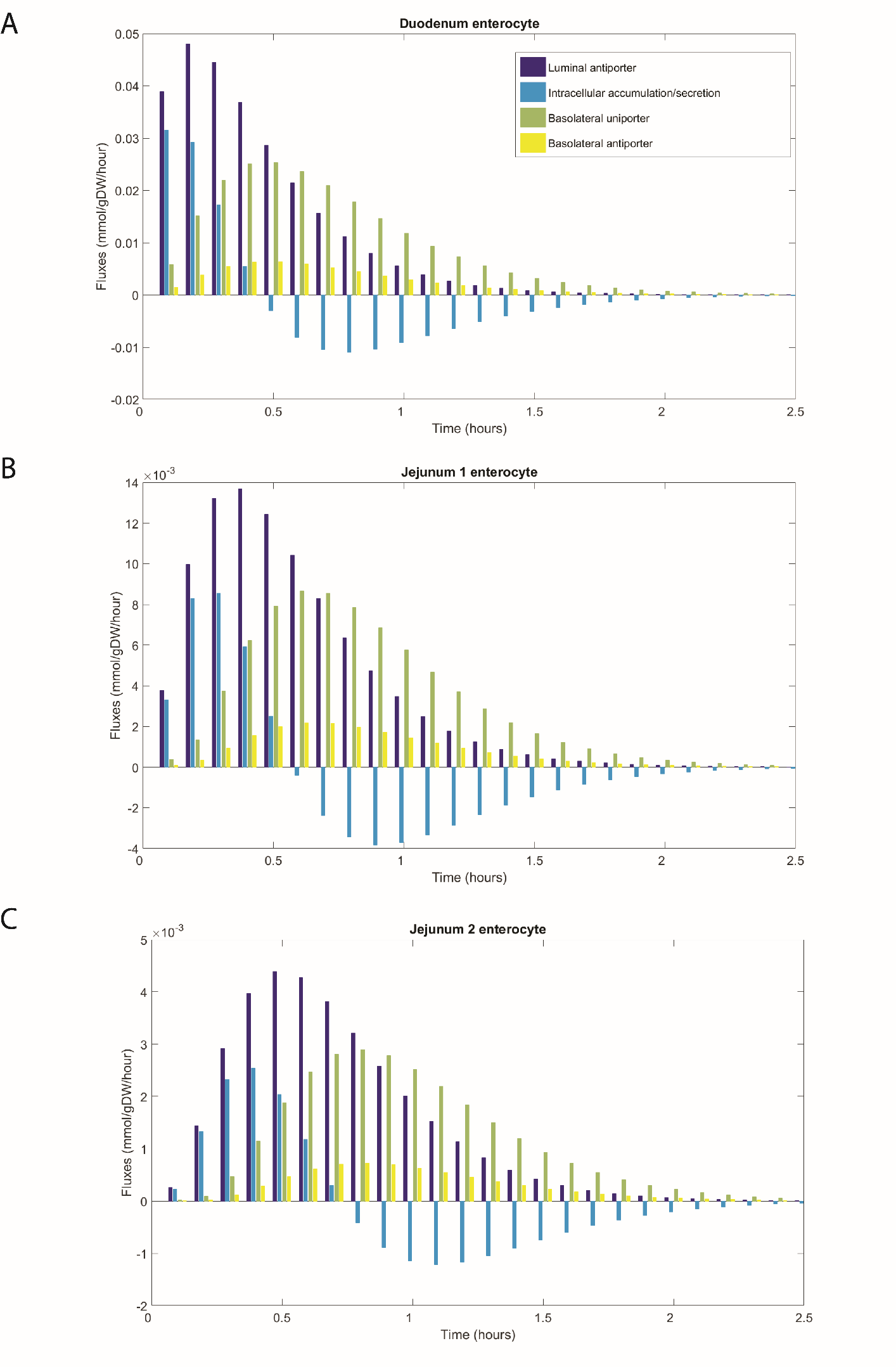


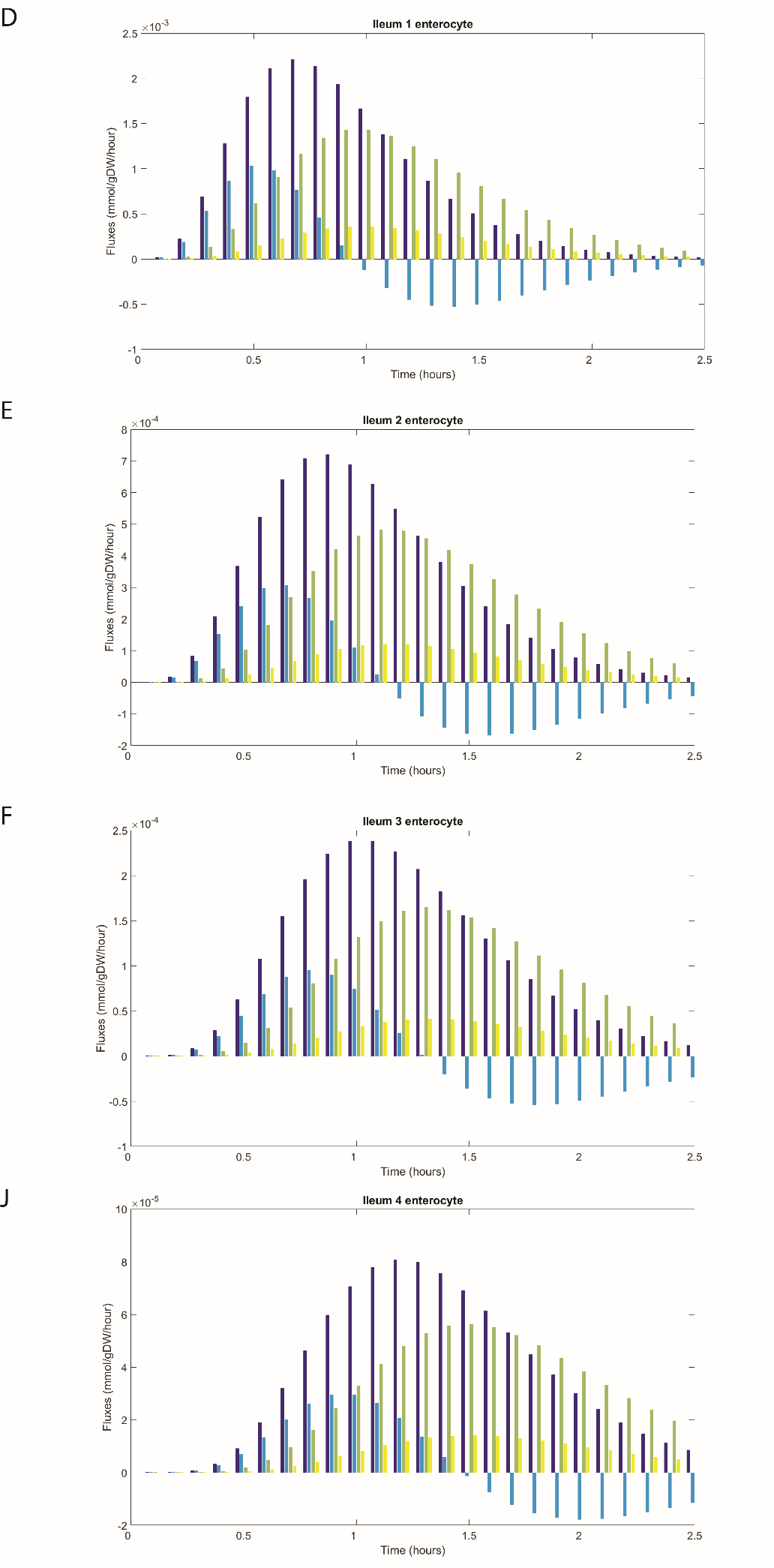


**Figure S3** – Predicted flux values of the levodopa luminal antiporter (blue), cellular accumulation (turquoise), basolateral antiporter (green), and basolateral uniporter (yellow) in the seven small intestinal compartments, after per os administration of 200 mg of standard formulation of levodopa at the fasted state. For the intracellular accumulation (represented in the model with a reversible drain for levodopa (sink_levodopa)), positive fluxes refer to accumulation, while negative fluxes represent secretion. The conversion to mmol/gDW_enterovyte_/hour was done as described in section adding levodopa reactions to sIEC.


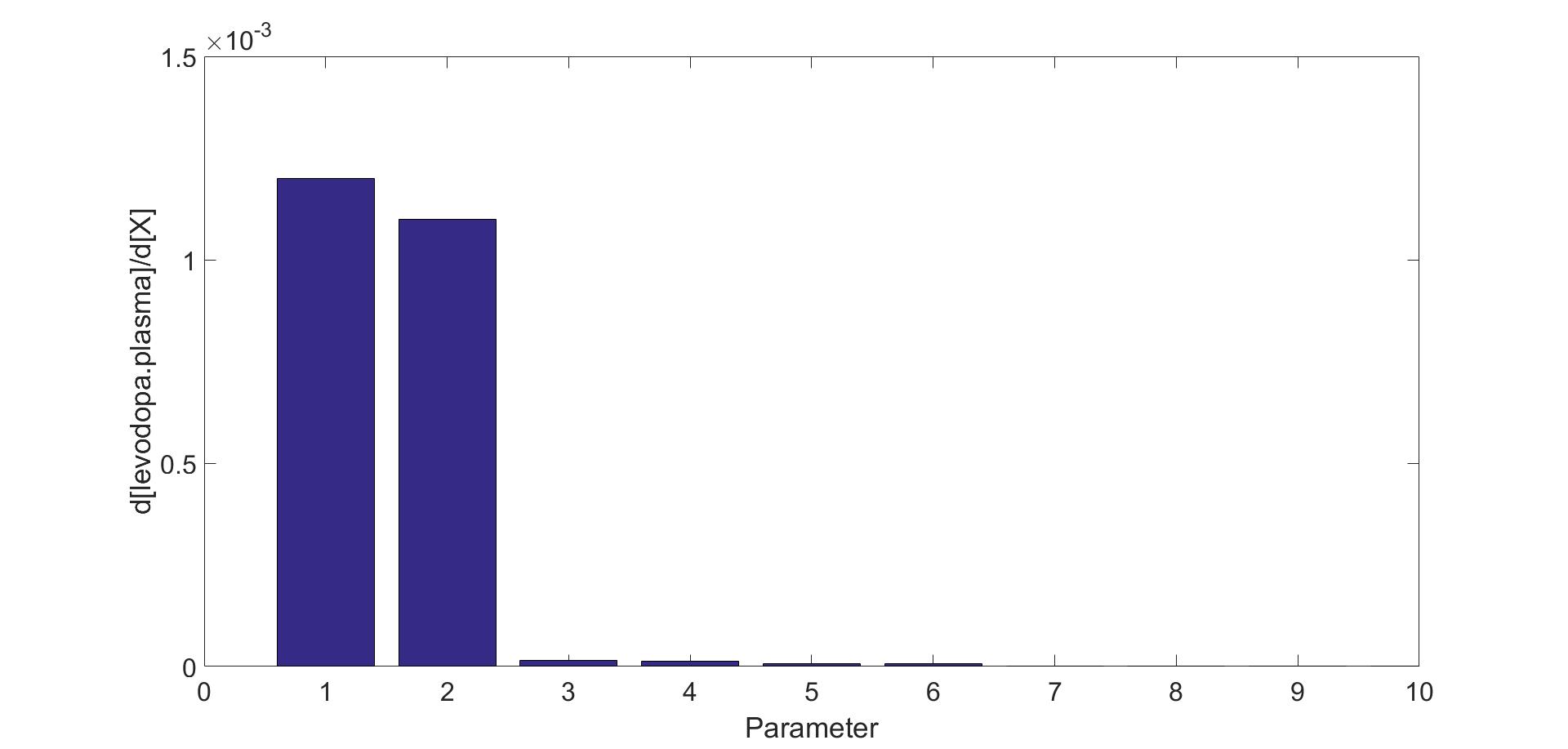


**Figure S4** – Whole body PBPK model parameter sensitivity analysis. Over 243 parameter, the 10 most influential absolute sensitivities are plotted. The first five parameters are described in Table S3.


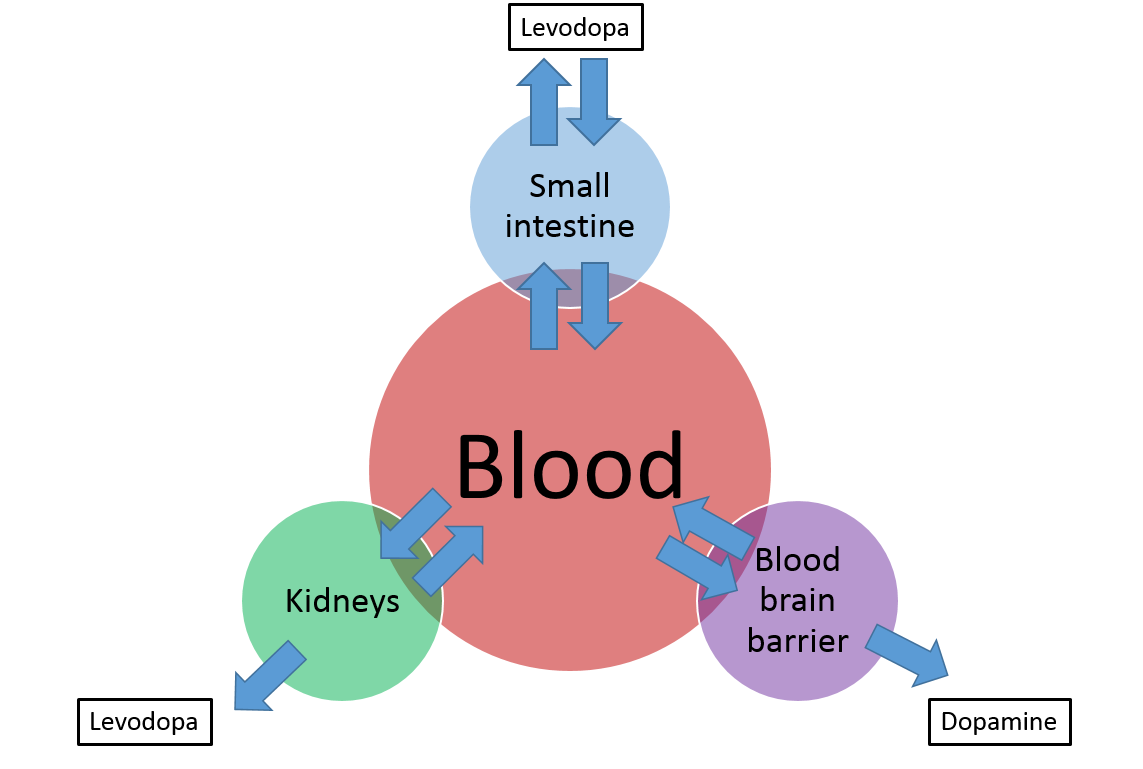


**Figure S5** – Schematic view of small intestine, kidney and blood brain barrier transport reactions integration. The small intestine is represents through the genome-scale sIEC model, while only the transport reactions from blood to the organ were modeled in the other sites of competition (e.g., kidneys and brain compartments).

**References:**

1. Peters SA. Evaluation of a generic physiologically based pharmacokinetic model for lineshape analysis. Clinical pharmacokinetics. 2008;47(4):261-75.

2. Agoram B, Woltosz WS, Bolger MB. Predicting the impact of physiological and biochemical processes on oral drug bioavailability. Advanced drug delivery reviews. 2001;50 Suppl 1:S41-67.

3. Camargo SM, Vuille-dit-Bille RN, Mariotta L, Ramadan T, Huggel K, Singer D, et al. The molecular mechanism of intestinal levodopa absorption and its possible implications for the treatment of Parkinson's disease. The Journal of pharmacology and experimental therapeutics. 2014;351(1):114-23.

4. Verrey F, Meier C, Rossier G, Kuhn LC. Glycoprotein-associated amino acid exchangers: broadening the range of transport specificity. Pflugers Archiv : European journal of physiology. 2000;440(4):503-12.

5. Orth JD, Thiele I, Palsson BO. What is flux balance analysis? Nature biotechnology. 2010;28(3):245-8.

6. Robertson DR, Higginson I, Macklin BS, Renwick AG, Waller DG, George CF. The influence of protein containing meals on the pharmacokinetics of levodopa in healthy volunteers. British journal of clinical pharmacology. 1991;31(4):413-7.

7. Nutt JG, Woodward WR, Hammerstad JP, Carter JH, Anderson JL. The "on-off" phenomenon in Parkinson's disease. Relation to levodopa absorption and transport. The New England journal of medicine. 1984;310(8):483-8.

8. Contin M, Martinelli P. Pharmacokinetics of levodopa. Journal of neurology. 2010;257(Suppl 2):S253-61.

9. Knox C, Law V, Jewison T, Liu P, Ly S, Frolkis A, et al. DrugBank 3.0: a comprehensive resource for 'omics' research on drugs. Nucleic acids research. 2011;39(Database issue):D1035-41.

10. Martinelli P, Contin M, Scaglione C, Riva R, Albani F, Baruzzi A. Levodopa pharmacokinetics and dyskinesias: are there sex-related differences? Neurological sciences : official journal of the Italian Neurological Society and of the Italian Society of Clinical Neurophysiology. 2003;24(3):192-3.

11. Lennernas H, Nilsson D, Aquilonius SM, Ahrenstedt O, Knutson L, Paalzow LK. The effect of L-leucine on the absorption of levodopa, studied by regional jejunal perfusion in man. British journal of clinical pharmacology. 1993;35(3):243-50.
