# Supplementary material for: Model-based dietary optimization for late-stage, levodopa-treated, Parkinson’s disease patients
Source: NPJ Syst Biol Appl. 2016 Jun 16;2:16013–. doi: 10.1038/npjsba.2016.13 (PMC5516849; doi:10.1038/npjsba.2016.13)
Supplement: Supplementary Table S1 [file npjsba201613-s2.doc]

**Table S1 – Ordinary differential equation-based levodopa pharmacokinetic model.**

| Equation name  Levodopa concentration in: | Description | Equation | Parameter description |
| --- | --- | --- | --- |
| Stomach transit compartment (tc) | Levodopa transit in stomach | 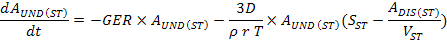  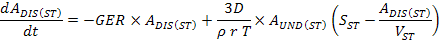 | where 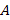 is the amount of the drug, S is solubility, UND and DIS refer to undissolved and dissolved, 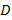 the diffusion coefficient of levodopa,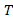 the diffusion layer thickness, 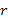 the drug particle density, 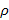 the effective drug particle radius ,and 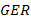 is the gastric emptying rate constant. |
| Duodenum tc | Levodopa transit in duodenum | 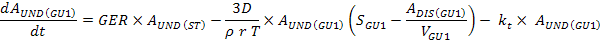  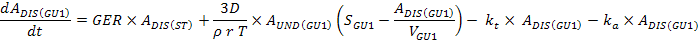 | , where 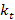 is the transit rate, and is 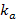 the absorption rate in the duodenum. |
| Jejunum to ileum tc | Levodopa transit from jejunum to ileum | 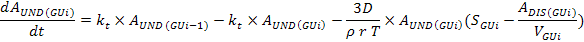  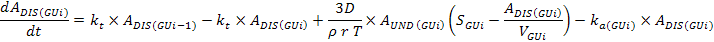  , where i = 2 to 7. | The absorption rate was assumed to be equal in all small intestine compartments as demonstrated *in vivo* (1). |
| Colon tc | Levodopa transit in colon | 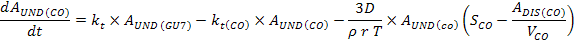  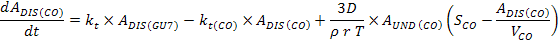 | ,where 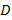 the diffusion coefficient of levodopa,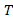 the diffusion layer thickness, 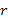 the drug particle density, 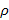 the effective drug particle radius ,and 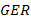 is the gastric emptying rate constant. |
| Small intestine enterocytes |  | 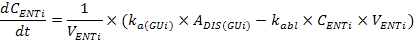  , where i = 2 to 7. |  |
| Non-eliminating organs (brain, spleen, pancreas, heart, muscle, adipose, skin, bone, thymus) | Levodopa concentration in non-eliminating organs | 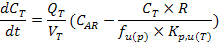 | , where T represents all non-eliminating organs. |
| Liver | Levodopa concentration in liver | 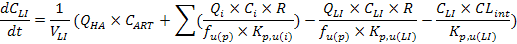 | ,where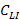 is the concentration of levodopa in the liver, 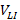 is the volume of the liver ,and i = gut, pancreas, spleen, and stomach. |
| Kidneys | Levodopa concentration in kidneys | 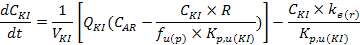 | ,where 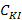 is the concentration of levodopa in the kidneys, 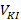 is the volume of the lungs ,and 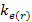 represents the renal elimination rate. |
| Stomach | Levodopa concentration in stomach | 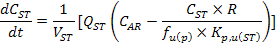 | , where 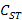 is the concentration of levodopa in the stomach, and 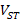 is the volume of the stomach. |
| Venous blood | Levodopa concentration in venous blood | 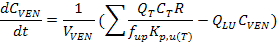 | , where T represents all organs connected to the venous circulation, and 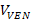 is the volume of the venous blood. |
| Arterial blood | Levodopa concentration in arterial blood | 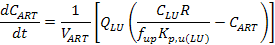 | , where 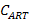is the concentration of levodopa in the arterial blood, and 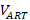 is the volume of the arterial blood. |
| Lungs | Levodopa concentration in lungs | 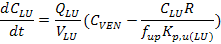 | , where 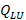is the blood flow rate in the lungs, 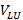 is the volume of the lungs, 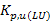 is the unbound tissue partition coefficient of the lungs, and 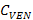 is the levodopa concentration in venous blood. |
| Gut | Levodopa concentration in gut | 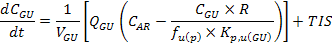  Total intestinal secretion (TIS)  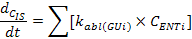  Total intestinal absorption (TIA)  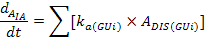 | , where 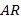 is arterial blood, 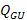 is the blood flow rate in the gut, 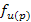is the fraction unbound in the plasma, R the blood to plasma partition coefficient, and 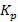 is the unbound tissue partition coefficient. |

The equations describing levodopa kinetics in the different organs were kept the same as in (2), elimination was assumed to occur completely in the kidneys. The human physiology parameters were set as in (2) and the drug specific parameters were estimated with healthy volunteers pharmacokinetics data (Table S1).

**References:**

1. Camargo SM, Vuille-dit-Bille RN, Mariotta L, Ramadan T, Huggel K, Singer D, et al. The molecular mechanism of intestinal levodopa absorption and its possible implications for the treatment of Parkinson's disease. The Journal of pharmacology and experimental therapeutics. 2014;351(1):114-23.

2. Peters SA. Evaluation of a generic physiologically based pharmacokinetic model for lineshape analysis. Clinical pharmacokinetics. 2008;47(4):261-75.
