# Supplementary material for: Model-based dietary optimization for late-stage, levodopa-treated, Parkinson’s disease patients
Source: NPJ Syst Biol Appl. 2016 Jun 16;2:16013–. doi: 10.1038/npjsba.2016.13 (PMC5516849; doi:10.1038/npjsba.2016.13)
Supplement: Supplementary Table S2 [file npjsba201613-s3.doc]

**Tables:**

**Table S2** – Whole body generic PBPK model parameter estimation from curve fitting.

| **Parameter** | **Model constraints** | **Estimated value** | **Literature value** |
| --- | --- | --- | --- |
| **Molecular weight** | 197.18 g/mol | Fixed | 197.18 g/mol^1^ (1) |
| **Blood plasma partition coefficient** | > 0 | 0.57 | - |
| **Clearance** | 0.5-5 l/kg/h | 0.95 l/kg/h | 0.55-1.38 l/kg/h (2) |
| **Log of permeability** | < 0 | -4.9 | -2.39^1^ |
| **Unbound fraction** | 0.6-0.95 | 0.95 | 0.6-0.95^1^ |
| **Effective luminal intestinal permeability** | 0-5 cm/hour | 1.8 cm/hour | 1.22 cm/hour (3) |
| **Distribution factor** | > 0 | 1.24 | - |
| **Effective basolateral intestinal permeability** | 0-5 cm/hour | 3.6 cm/hour | ≥ Effective luminal intestinal permeability (4) |

The estimated parameters are within the reported biological interval. The clearance regroups the overall elimination from the body in the eliminating organs (brain, kidneys, liver). The parameters units are mentioned and dimensionless otherwise.

**References:**

1. Knox C, Law V, Jewison T, Liu P, Ly S, Frolkis A, et al. DrugBank 3.0: a comprehensive resource for 'omics' research on drugs. Nucleic acids research. 2011;39(Database issue):D1035-41.

2. Martinelli P, Contin M, Scaglione C, Riva R, Albani F, Baruzzi A. Levodopa pharmacokinetics and dyskinesias: are there sex-related differences? Neurological sciences : official journal of the Italian Neurological Society and of the Italian Society of Clinical Neurophysiology. 2003;24(3):192-3.

3. Lennernas H, Nilsson D, Aquilonius SM, Ahrenstedt O, Knutson L, Paalzow LK. The effect of L-leucine on the absorption of levodopa, studied by regional jejunal perfusion in man. British journal of clinical pharmacology. 1993;35(3):243-50.

4. Agoram B, Woltosz WS, Bolger MB. Predicting the impact of physiological and biochemical processes on oral drug bioavailability. Advanced drug delivery reviews. 2001;50 Suppl 1:S41-67.

^1^ <http://www.drugbank.ca/drugs/DB01235>
