# Supplementary material for: Model-based dietary optimization for late-stage, levodopa-treated, Parkinson’s disease patients
Source: NPJ Syst Biol Appl. 2016 Jun 16;2:16013–. doi: 10.1038/npjsba.2016.13 (PMC5516849; doi:10.1038/npjsba.2016.13)
Supplement: Supplementary Table S3 [file npjsba201613-s4.doc]

**Table S3 – Reactions added to the sIEC model ordered by affinity of amino acids to the corresponding transporter.**

| **Luminal antiport reactions** | **GPR (Entrez ID)** |
| --- | --- |
| 34dhphe[u] + ala_L[c] 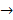 34dhphe[c] + ala_L[u] | 11136 and 6519  (1) |
| 34dhphe[u] + leu_L[c] 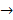 34dhphe[c] + leu_L[u] |
| 34dhphe[u] + arg_L[c] 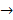 34dhphe[c] + arg_L[u] |
| **Basolateral antiport reactions** | **GPR (Entrez ID)** |
| 34dhphe[c] + tyr_L[e] 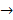 34dhphe[e] + tyr_L[c] | 23428 and 6520  (1) |
| 34dhphe[c] + trp_L[e] 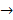 34dhphe[e] + trp_L[c] |
| 34dhphe[c] + phe_L[e] 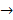 34dhphe[e] + phe_L[c] |
| 34dhphe[c] + thr_L[e] 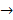 34dhphe[e] + thr_L[c] |
| 34dhphe[c] + ile_L[e] 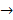 34dhphe[e] + ile_L[c] |
| 34dhphe[c] + cys_L[e] 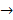 34dhphe[e] + cys_L[c] |
| 34dhphe[c] + ser_L[e] 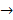 34dhphe[e] + ser_L[c] |
| 34dhphe[c] + val_L[e] 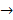 34dhphe[e] + val_L[c] |
| 34dhphe[c] + leu_L[e] 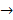 34dhphe[e] + leu_L[c] |
| 34dhphe[c] + glu_L[e] 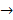 34dhphe[e] + glu_L[c] |
| 34dhphe[c] + ala_L[e] 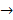 34dhphe[e] + ala_L[c] |
| 34dhphe[c] + his_L[e] 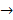 34dhphe[e] + his_L[c] |
| 34dhphe[c] + asp_L[e] 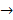 34dhphe[e] + asp_L[c] |
| 34dhphe[c] + met_L[e] 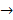 34dhphe[e] + met_L[c] |
| **Complementary luminal amino acids antiport reactions** | **GPR (Entrez ID)** |
| leu_L[u] + ala_L[c] 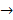 leu_L[c] + ala_L[u] | 11136 and 6519  (1) |
| leu_L[u] + arg_L[c] 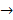 leu_L[c] + arg_L[u] |
| lys_L[u] + arg_L[c] 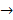 lys_L[c] + arg_L[u] |
| lcystin[u] + arg_L[c] 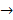 lcystin[c] + arg_L[u] |
| orn[u] + arg_L[c] 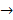 orn[c] + arg_L[u] |
| tyr_L[u] + ala_L[c] 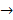 tyr_L[c] + ala_L[u] |
| tyr_L[u] + arg_L[c] 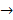 tyr_L[c] + arg_L[u] |
| ala_L[u] + arg_L[c] 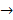 ala_L[c] + arg_L[u] |
| ala_L[u] + leu_L[c] 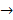 ala_L[c] + leu_L[u] |
| **Intracellular accumulation** | **References** |
| (Demand reaction) 34dhphe[c] => | (2) |
| **Luminal competition** | **References** |
| 0.42 34dhphe[u] + 0.1 lcystin[u] 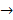 artefact[u] | (1, 2) |
| 0.38 34dhphe[u] + 0.1 arg_L[u] 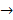 artefact[u] |
| 0.34 34dhphe[u] + 0.1 lys_L[u] 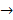 artefact[u] |
| 0.3 34dhphe[u] + 0.1 leu_L[u] 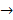 artefact[u] |
| 0.26 34dhphe[u] + 0.1 tyr_L[u] 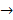 artefact[u] |
| 0.22 34dhphe[u] + 0.1 ala_L[u] 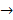 artefact[u] |
| 0.18 34dhphe[u] + 0.1 orn[u] 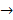 artefact[u] |
| (Demand reaction) artefact[u] => |
| **Trans-stimulation with basolateral amino acids** | **References** |
| (Demand reaction) 34dhphe[c] * 0.77 => | (2) |

The basolateral uniport reaction exits already in the original sIEC, its lower bound was set to zero to avoid free diffusion back in the cell from the basolateral side. 34dhphe represents levodopa and artefact represents a dummy molecule that accounts for the luminal loss of levodopa in the presence of amino acids. The stoichiometric coefficients of luminal competition were inferred from reported in vitro experiments and represent the percentage of loss of levodopa. u, c and e stand for lumen, cytoplasm and blood, respectively. GPR stands for gene protein reaction.

**References:**

1. Verrey F, Meier C, Rossier G, Kuhn LC. Glycoprotein-associated amino acid exchangers: broadening the range of transport specificity. Pflugers Archiv : European journal of physiology. 2000;440(4):503-12.

2. Camargo SM, Vuille-dit-Bille RN, Mariotta L, Ramadan T, Huggel K, Singer D, et al. The molecular mechanism of intestinal levodopa absorption and its possible implications for the treatment of Parkinson's disease. The Journal of pharmacology and experimental therapeutics. 2014;351(1):114-23.
