# Supplementary material for: Model-based dietary optimization for late-stage, levodopa-treated, Parkinson’s disease patients
Source: NPJ Syst Biol Appl. 2016 Jun 16;2:16013–. doi: 10.1038/npjsba.2016.13 (PMC5516849; doi:10.1038/npjsba.2016.13)
Supplement: Supplementary Table S4 [file npjsba201613-s5.doc]

**Table S4 – Top 5 ranking parameter sensitivities.**

| **Rank** | **Parameter** | **Description** |
| --- | --- | --- |
| 1 | intestlo | Intestinal loss of levodopa. |
| 2 | stomlo | Stomach loss of levodopa. |
| 3 | 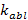 | Secretion of levodopa in the portal vein through the basolateral membrane of enterocyte. |
| 4 | 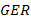 | Gastric emptying rate. |
| 5 | 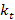 | Small intestine transit rate constant. |

The parameters are represented with their model designation and a description of their biological properties.
