# Supplementary material for: Model-based dietary optimization for late-stage, levodopa-treated, Parkinson’s disease patients
Source: NPJ Syst Biol Appl. 2016 Jun 16;2:16013–. doi: 10.1038/npjsba.2016.13 (PMC5516849; doi:10.1038/npjsba.2016.13)
Supplement: Supplementary Table S5 [file npjsba201613-s6.doc]

**Table S5 – Levodopa exchange substrate in the kidney and in the blood brain barrier.**

| **Compartment** | **Substrates in**  **(ordered by affinity for the transporter)** | **Substrates out** | **GPR (Entrez ID)** | **References** |
| --- | --- | --- | --- | --- |
| Brain | leu,his,ile,phe,tyr,trp,  val,met,gln,levodopa | leu | 855653 | (1) |
| Kidney | tyr,trp,phe,thr,ile,cys,ser,  val,leu,gln,ala,his,asn,met,  gly,levodopa | phe,ile,leu | 7462 | (1) |

The substrates are ordered by affinity to the transporter. GPR stands for gene protein reaction.

**References:**

1. Verrey F, Meier C, Rossier G, Kuhn LC. Glycoprotein-associated amino acid exchangers: broadening the range of transport specificity. Pflugers Archiv : European journal of physiology. 2000;440(4):503-12.
