# Supplementary material for: Model-based dietary optimization for late-stage, levodopa-treated, Parkinson’s disease patients
Source: NPJ Syst Biol Appl. 2016 Jun 16;2:16013–. doi: 10.1038/npjsba.2016.13 (PMC5516849; doi:10.1038/npjsba.2016.13)
Supplement: Supplementary Table S6 [file npjsba201613-s7.doc]

**Table S6 – Constraints subjected to a three organ model accounting for levodopa absorption, elimination, metabolism and competition with amino acids**.

| **Added reactions and affinity coefficients** | **Constraints**  **(ub, lb)** |
| --- | --- |
| Small intestine lumen : 34dhphe[u] => | -15 |
| {amino acid}_L[u] => | -3 |
| 34dhphe[u] 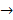 34dhphe[elim] (non-absorbed fraction in the gut) | 5 |
| **Blood brain barrier reactions** | |
| Blood brain barrier : 34dhphe[e] 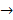 34dhphe[bbb] | unc |
| (Demand reaction) 34dhphe[bbb] => | unc |
| 0.23 34dhphe[e] + 1 leu_L[e] 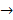 0.23 34dhphe[bbb] + 1 leu_L[bbb] | unc |
| 0.26 34dhphe[e] + 1 his_L[e] 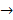 0.26 34dhphe[bbb] + 1 his_L[bbb] | unc |
| 0.29 34dhphe[e] + 1 ile_L[e] 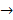 0.29 34dhphe[bbb] + 1 ile_L[bbb] | unc |
| 0.33 34dhphe[e] + 1 phe_L[e] 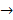 0.33 34dhphe[bbb] + 1 phe_L[bbb] | unc |
| 0.36 34dhphe[e] + 1 tyr_L[e] 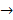 0.36 34dhphe[bbb] + 1 tyr_L[bbb] | unc |
| 0.39 34dhphe[e] + 1 trp_L[e] 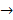 0.39 34dhphe[bbb] + 1 trp_L[bbb] | unc |
| 0.43 34dhphe[e] + 1 val_L[e] 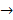 0.43 34dhphe[bbb] + 1 val_L[bbb] | unc |
| 1 34dhphe[e] + 1 thr_L[e] 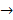 1 34dhphe[bbb] + 1 thr_L[bbb] | unc |
| 1 34dhphe[e] + 1 cys_L[e] 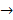 1 34dhphe[bbb] + 1 cys_L[bbb] | unc |
| 1 34dhphe[e] + 1 ser_L[e] 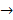 1 34dhphe[bbb] + 1 ser_L[bbb] | unc |
| 0.58 34dhphe[e] + 1 glu_L[e] 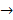 0.58 34dhphe[bbb] + 1 glu_L[bbb] | unc |
| 1 34dhphe[e] + 1 ala_L[e] 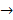 1 34dhphe[bbb] + 1 ala_L[bbb] | unc |
| 1 34dhphe[e] + 1 asn_L[e] 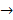 1 34dhphe[bbb] + 1 asn_L[bbb] | unc |
| 1 34dhphe[e] + 1 gln_L[e] 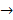 1 34dhphe[bbb] + 1 gln_L[bbb] | unc |
| 1 34dhphe[e] + 1 gly_L[e] 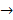 1 34dhphe[bbb] + 1 gly_L[bbb] | unc |
| 1 34dhphe[e] + 1 pro_L[e] 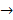 1 34dhphe[bbb] + 1 pro_L[bbb] | unc |
| 1 34dhphe[e] + 1 lys_L[e] 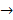 1 34dhphe[bbb] + 1 lys_L[bbb] | unc |
| 1 34dhphe[e] + 1 arg_L[e] 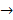 1 34dhphe[bbb] + 1 arg_L[bbb] | unc |
| 1 34dhphe[e] + 1 asp_L[e] 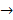 1 34dhphe[bbb] + 1 asp_L[bbb] | unc |
| 0.5 34dhphe[e] + 1 met_L[e] 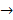 0.5 34dhphe[bbb] + 1 met_L[bbb] | unc |
| **Kidney reactions** | |
| Kidney : 34dhphe[e] 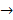 34dhphe[ku] | 3 (30% of absorbed fraction) |
| 0.5 34dhphe[ku] + 1 tyr_L[ku] 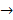 0.5 34dhphe[k] + 1 tyr_L[k] | unc |
| 0.5 34dhphe[ku] + 1 trp_L[ku] 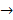 0.5 34dhphe[k] + 1 trp_L[k] | unc |
| 0.5 34dhphe[ku] + 1 phe_L[ku] 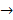 0.5 34dhphe[k] + 1 phe_L[k] | unc |
| 0.6 34dhphe[ku] + 1 thr_L[ku] 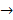 0.6 34dhphe[k] + 1 thr_L[k] | unc |
| 0.63 34dhphe[ku] + 1 ile_L[ku] 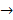 0.63 34dhphe[k] + 1 ile_L[k] | unc |
| 0.66 34dhphe[ku] + 1 cys_L[ku] 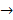 0.66 34dhphe[k] + 1 cys_L[k] | unc |
| 0.69 34dhphe[ku] + 1 ser_L[ku] 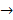 0.69 34dhphe[k] + 1 ser_L[k] | unc |
| 0.73 34dhphe[ku] + 1 val_L[ku] 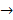 0.73 34dhphe[k] + 1 val_L[k] | unc |
| 0.76 34dhphe[ku] + 1 leu_L[ku] 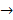 0.76 34dhphe[k] + 1 leu_L[k] | unc |
| 0.79 34dhphe[ku] + 1 glu_L[ku] 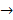 0.79 34dhphe[k] + 1 glu_L[k] | unc |
| 0.83 34dhphe[ku] + 1 ala_L[ku] 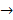 0.83 34dhphe[k] + 1 ala_L[k] | unc |
| 0.86 34dhphe[ku] + 1 his_L[ku] 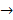 0.86 34dhphe[k] + 1 his_L[k] | unc |
| 0.89 34dhphe[ku] + 1 asn_L[ku] 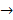 0.89 34dhphe[k] + 1 asn_L[k] | unc |
| 0.93 34dhphe[ku] + 1 gln_L[ku] 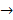 0.93 34dhphe[k] + 1 gln_L[k] | unc |
| 1 34dhphe[ku] + 1 gly_L[ku] 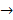 1 34dhphe[k] + 1 gly_L[k] | unc |
| 1 34dhphe[ku] + 1 pro_L[ku] 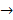 1 34dhphe[k] + 1 pro_L[k] | unc |
| 1 34dhphe[ku] + 1 lys_L[ku] 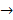 1 34dhphe[k] + 1 lys_L[k] | unc |
| 1 34dhphe[ku] + 1 arg_L[ku] 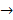 1 34dhphe[k] + 1 arg_L[k] | unc |
| 1 34dhphe[ku] + 1 asp_L[ku] 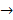 1 34dhphe[k] + 1 asp_L[k] | unc |
| 1 34dhphe[ku] + 1 met_L[ku] 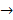 1 34dhphe[k] + 1 met_L[k] | unc |
| **Blood reactions** | |
| (Demand reaction) ala_L => | -100 |

While the small intestine has been represented by the genome scale model, only levodopa transport reactions were modeled for the kidneys and blood brain barrier. Amino acids that do not compete with levodopa have a stoichiometric coefficient of 1, amino acids that compete have a coefficient < 1, the higher the affinity for the transporter the lower the coefficient. 20 models were generated, one for every combination of one amino acid and levodopa. ku stands for kidney lumen, bbb for blood brain barrier ,and unc for unconstrained.
